# Importance of T, NK, CAR T and CAR NK Cell Metabolic Fitness for Effective Anti-Cancer Therapy: A Continuous Learning Process Allowing the Optimization of T, NK and CAR-Based Anti-Cancer Therapies

**DOI:** 10.3390/cancers14010183

**Published:** 2021-12-30

**Authors:** Adrien Krug, Adriana Martinez-Turtos, Els Verhoeyen

**Affiliations:** 1 Université Côte d’Azur, INSERM, C3M, 06204 Nice, France; adrien.krug@etu.univ-cotedazur.fr (A.K.); Adrianna.MARTINEZ-TURTOS@univ-cotedazur.fr (A.M.-T.); 2 CIRI, Université de Lyon, INSERM U1111, ENS de Lyon, Université Lyon1, CNRS, UMR 5308, 69007 Lyon, France

**Keywords:** T cell, NK cell, CAR T cell, CAR NK cell, metabolism, glycolysis, OXPHOS, immunotherapy, cancer therapy

## Abstract

**Simple Summary:**

Cancer treatments are evolving at a very rapid pace. Some of the most novel anti-cancer medicines under development rely on the modification of immune cells in order to transform them into potent tumor-killing cells. However, the tumor microenvironment (TME) is competing for nutrients with these harnessed immune cells and therefore paralyzes their metabolic effective and active anti-cancer activities. Here we describe strategies to overcome these hurdles imposed on immune cell activity, which lead to therapeutic approaches to enhance metabolic fitness of the patient’s immune system with the objective to improve their anti-cancer capacity.

**Abstract:**

Chimeric antigen receptor (CAR) T and CAR NK cell therapies opened new avenues for cancer treatment. Although original successes of CAR T and CAR NK cells for the treatment of hematological malignancies were extraordinary, several obstacles have since been revealed, in particular their use for the treatment of solid cancers. The tumor microenvironment (TME) is competing for nutrients with T and NK cells and their CAR-expressing counterparts, paralyzing their metabolic effective and active states. Consequently, this can lead to alterations in their anti-tumoral capacity and persistence in vivo. High glucose uptake and the depletion of key amino acids by the TME can deprive T and NK cells of energy and building blocks, which turns them into a state of anergy, where they are unable to exert cytotoxic activity against cancer cells. This is especially true in the context of an immune-suppressive TME. In order to re-invigorate the T, NK, CAR T and CAR NK cell-mediated antitumor response, the field is now attempting to understand how metabolic pathways might change T and NK responses and functions, as well as those from their CAR-expressing partners. This revealed ways to metabolically rewire these cells by using metabolic enhancers or optimizing pre-infusion in vitro cultures of these cells. Importantly, next-generation CAR T and CAR NK products might include in the future the necessary metabolic requirements by improving their design, manufacturing process and other parameters. This will allow the overcoming of current limitations due to their interaction with the suppressive TME. In a clinical setting, this might improve their anti-cancer effector activity in synergy with immunotherapies. In this review, we discuss how the tumor cells and TME interfere with T and NK cell metabolic requirements. This may potentially lead to therapeutic approaches that enhance the metabolic fitness of CAR T and CAR NK cells, with the objective to improve their anti-cancer capacity.

## 1. T Cell and CAR T Cell Metabolism Plays a Major Role in Anti-Cancer Immunity

### 1.1. T Cell Metabolism in a “Healthy” Environment

T cells are major components of the adaptive immune system. CD4+ cells, as well as CD8+ T cells, function as effectors of the immune system. T cells continuously screen lymphoid and peripheral tissues such as the spleen and lymph nodes for antigens (peptides or lipids) that are presented by the major histocompatibility complex (MHC) of antigen-presenting cells (APCs). APCs include macrophages, dendritic cells and B cells. When T cells recognize a specific antigen from pathogens or tumor cells through their TCR, they start to expand and migrate to the diseased tissues, where they exert their effector functions by killing infected or malignant cells. In order to perform these effector functions, T cells undergo complex molecular changes. Once the TCR is engaged, protein tyrosine kinases phosphorylate tyrosine residues situated in the cytoplasmic tail of the TCR, which then binds to various signaling molecules that activate multiple transcription factors that transform naïve T cells into effector T cells [1].

This requires an extensive “metabolic reprogramming” of T cells in order to provide the energy and building blocks for their clonal expansion and to ensure their anti-cancer activity. Some of the earlier studies on immunometabolism of human T cells were published in the context of HIV. HIV-1 mainly infects CD4+ T cells and accumulation. The evidence has brought to light the association between T cell metabolism reprogramming and HIV-1 pathogenesis [2,3]. Recently, metabolic reprogramming of T cells was proposed as an approach for HIV cure and HIV reservoir eradication [4]. Resting T cells in our bloodstream rely mainly on oxidative phosphorylation (OXPHOS) and fatty acid oxidation in the mitochondria to generate enough ATP, which sustains their homeostasis. Upon antigen encounter, they rapidly start to increase glucose and amino acid (aa) uptake, which alters their metabolism to glycolysis, by increasing the activity of multiple kinases (Phosphoinositide 3-kinase (PI3K)/Protein kinase B (AKT)/mammalian target of rapamycin (mTOR) [5,6,7,8,9]. For example, activation of mTOR promotes glycolysis through the upregulation of the major regulator c-Myc and hypoxia-inducible factor 1 α (HIF1α) [10,11]. Glucose uses mainly the glucose transporter 1 (GLUT1) transporter for its uptake, whereas aa such as glutamine uses large amino acid transporter 1 (LAT1), serotonin N-acetyltransferase (SNAT-1,-2) and ASC amino acid transporter 2 (ASCT2) [12,13,14]. In human and mouse T cells, antigen exposure that requires a co-stimulatory signal through CD28 [15,16] results in activation of the central metabolic regulator mTOR, which boosts the GLUT1 transporter ensuring increased glucose uptake [17,18,19].

Glucose is first metabolized into pyruvate, which according to the T cell activation state, follows a different metabolic pathway. Resting T cells convert pyruvate to acetyl-CoA for mitochondrial respiration. Interleukin 7 (IL-7) is the main survival cytokine required for the maintenance of these cells as it upregulates GLUT1 for glucose uptake [20,21]. In contrast, antigen-stimulated T cells increase their glucose uptake 18-fold, compared to resting T cells [22], and preferentially convert the resulting pyruvate into lactate, which is then secreted via the monocarboxylate lactate transporters (MCT), MCT1 and MCT4 [16,23,24]. Of note, pyruvate can also be directly imported by the MCTs [25]. This process is called “aerobic glycolysis” and permits a more rapid metabolism of incoming glucose to pyruvate. This occurs by rapid regeneration of the cofactor NAD+, while at the same time, provides many precursors for aa, protein and lipid synthesis that are all required by rapidly dividing cells. Additionally, increased glycolytic flux also increases the expression of effector molecules and requires high rate glycolytic enzymes, such as GAPDH, which is then unable to exercise non-glycolytic functions, including interferon γ (INFγ) inhibition at the mRNA level [26]. In parallel, activated murine and human T cells also continue to some extent their mitochondrial metabolism because some of the pyruvates from glycolysis can enter into the mitochondria and is converted into acetyl-CoA, which enters the tricarboxylic acid (TCA) cycle to produce carbon dioxide and water, but also drives the electron transport chain to produce ATP and reactive oxygen species (ROS) [27,28,29].

An essential aa such as glutamine is required for T cell proliferation [13,16,27]. Activated T cells upregulate glutamine transporters and glutaminolytic enzymes, which metabolize glutamine to α-ketoglutarate that fuels the TCA cycle [16]. T cell proliferation is not only sustained by ATP generation but also by ROS production, which stabilizes effector molecule expression and redox homeostasis in murine T cells [29,30]. Moreover, inhibition of the mitochondrial transport chain in murine T cells and hematopoietic stem cells renders them functionally incompetent in vivo, underlining the importance of mitochondrial function in these cells [29,31]. Importantly, it was recently revealed that intermediate products of the TCA cycle are implicated in histone modification, thereby modulating gene transcription and function of several genes, important for T cell fitness [30,32,33]. It is important to note that in the activation phase, effector T cells use mainly aerobic glycolysis. However, once they become memory T cells, they adapt again to OXPHOS metabolism, as shown in the context of murine T cells [34]. Subsequently, once they are repeatedly challenged, murine T cells, as well as human T cells, rapidly undergo a reactivation by adapting their metabolism [35,36].

We further focus on the T cell immunometabolism and function in the context of cancer and cancer treatment, focusing on chimeric antigen receptor (CAR) T cell immunotherapy.

### 1.2. T Cells Metabolism in the “Tumor” Microenvironment (Figure 1)

**Figure 1:**
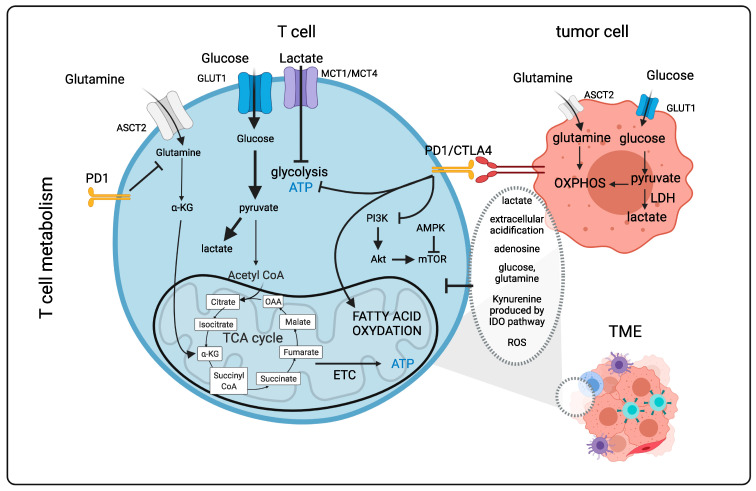
T cell metabolism in the tumor microenvironment. Naïve T cells rely mainly on oxidative metabolism. Following activation with an antigen, T cells switch to a glycolytic metabolism by activation of the mTOR pathway. This metabolic program supports effector T cell functions. If antigen stimulation persists long term, such as in the tumor environment, inhibitory receptors such as PD1 and CTLA4 can rewire T cell metabolism by reducing glycolysis and glutaminolysis, which weakens effector functions. Other factors in the TME contributing to the exhausted state of T cells include low levels of oxygen, low levels of tryptophan metabolized into kynurenine by IDO, low levels of arginine, high levels of lactate and resulting acidification and strong competition of T cells with cancer cells for glucose and glutamine. PD1: Programmed cell death 1; CTLA4: cytotoxic T-lymphocyte-associated protein 4; ASCT2: ASC amino-acid transporter 2; GLUT1: glucose transporter 1; OAA: Oxaloacetate; α-KG: α-Ketoglutarate; AMPK: Adenosine monophosphate kiinase; LDH: Lactate dehydrogenase; TME: Tumor microenvironment; Akt: Protein kinase B; mTOR: mammalian target of rapamycin; ATP: Adenosine triphosphate; ROS: Reactive oxygen species; PI3K: Phosphoinositide 3-kinase; IDO: Indoleamine-pyrrole 2,3-dioxygenase; ETC: Electron transport chain; TCA: Tricarboxylic acid; CoA: Coenzyme A. Figure generated with Biorender.com (accessed on 15 November 2021).

T cell therapies are very effective for hematopoietic malignancies [37,38,39,40]. However, for solid tumors, this is not the case since the metabolic environment can be very immunosuppressive, reverting murine and human T cells into a nonfunctional exhausted phenotype [41,42,43]. Uncovering these immune-metabolic hurdles will be essential for a more rational design of future cancer therapies. Acidity, low oxygen levels (hypoxia), suppressive metabolites and low nutrient availability encountered in the tumor microenvironment (TME) can severely suppress therapeutic T cell effector function. This was previously not considered in the context of clinical trials. However, it now represents a major concern in the field of immunotherapy and has encouraged an extensive field of research. Tumor cells rely strongly on glycolysis for their energy (ATP) production. Strangely enough, it has become clear that tumors cells still have the capacity to use OXPHOS, which is a metabolic pathway that generates more ATP. However, cancer cells use mainly glucose for fueling glycolysis, even in the presence of sufficient oxygen. This phenomenon, called aerobic glycolysis, was discovered by Warburg (Warburg effect; [44,45,46,47,48]). Glycolysis leads to strong tumor cell proliferation that depletes the TME of nutrients for the T infiltrating lymphocytes (TILs) and also produces immunosuppressive metabolites [26,49]. However, as indicated above, T cells can make a sudden switch in their metabolism from oxidative phosphorylation to glycolysis in order to exert their effector function [6,26]. This actually means that activated/effector T cells adapt an anaerobic glycolytic metabolic program in a similar manner to highly proliferating cancer cells [5]. Thus, T cells and cancer cells are in continuous competition for the same nutrients to sustain themselves (Figure 1). Importantly, T cell metabolic requirements can vary with the type of solid tumor. Indeed, glucose uptake by tumors and the TME results in glucose deprivation for T cells. Additionally, the byproduct lactate is extremely immunosuppressive, which leads to the acidification of the tumor environment [50,51,52], weakening CD8+ T cell effector functions, proliferation and cytokine production both in the murine and human context [53,54]. In the TME, T cells are often subjected to hypoxia, leading to the inhibition of mitochondrial function, a decrease in reactive oxygen species (ROS) and ATP levels, which paralyze effector T cells both in the murine and human context [55].

Another important mechanism by which cancer cells inhibit T cell effector functions is through the expression of ligands to immune checkpoint molecules such as programmed cell death 1 (PD-1) and cytotoxic T lymphocyte-associated protein 4 (CTLA-4), which are highly upregulated on the surface of activated T cells. PD-1 and CTLA-4 engagement inhibit mTOR function through the protein phosphatase 2A (PP2A) and SH2-domain-containing tyrosine phosphatase 2 (SHP-S) signaling, respectively [56,57,58,59,60]. It was shown that that co-inhibitory immune checkpoint blockade (e.g., anti-PD-1, anti-CTLA-4) reduced glucose uptake by the tumor cells reinstating glucose availability for the T cells. This might, in part, explain the success of this kind of immunotherapy [61]. T-cell immunoglobulin and mucin domain-3 (TIM3), another co-inhibitory molecule, can activate mTOR; however, the mechanism by which this occurs has yet to be revealed [62]. In the TME, regulatory T cells and myeloid-derived suppressor cells (MDSCs) can secrete factors such as transforming growth factor β (TGFβ) and Indoleamine-pyrrole 2,3-dioxygenase (IDO), which reduce human T cell function by mTOR suppression [63]. Furthermore, IL10, IL35 and adenosine can severely affect human effector T cell activity [64].

In the TME, only low levels of amino acids remain available for T cells. For example, glutamine is a primary energy source for tumor cells; however, low levels of glutamine are detrimental for T cell activation and function [12,14]. The aa arginine is also essential for human T cell function and is strongly depleted from the TME because it also becomes consumed by the cancer cells [65,66,67].

TIL and chimeric antigen receptor (CAR) T cells are clearly subjected to the same challenges in the TME that inhibit effector T cell metabolism and function. Below, we introduce this novel immunotherapy consisting of presenting newly engineered CARs on T cells. In order to reveal how to improve immunotherapies by reactivating TILs as well as CAR T cells or protecting them against the hostile TME, we address the different components in the TME that contribute to the changes in T effector metabolism in more detail and how one could therapeutically interfere with these obstacles to revert the exhausted T cells or CAR T cells into potent anti-cancer effector T cells.

### 1.3. CAR T Cells for Anticancer Treatment: Latest Developments

In cancer, particularly in the context of hematological tumors, T cells are also used as a therapeutic tool. In this type of immunotherapy, the patient’s T cells are genetically engineered ex vivo to express a CAR that recognizes a specific antigen present on the surface of the tumor cells. After reinfusion of these cells in the patient’s circulation, the binding between the CAR T cells and the cancer cells induces a cytotoxic response. One example of this therapy currently being used in clinics is for the treatment of advanced B-cell lymphomas, resulting in complete remission in 30 to 40 % of the patients. Importantly, it needs to be emphasized that CAR T cells are used last in line as a therapeutic strategy to suppress tumor cells. This means that the T cells are often isolated from the patients after various treatments, including chemotherapy that can alter the metabolic phenotype of the T cells (mitochondrial damage and metabolic alterations) [68]. Therefore, metabolism is an important aspect in the conception and the anti-tumoral activity of a CAR T cell.

#### 1.3.1. Continuous Improvements in the CAR Design to Stir CAR T Cell Metabolism (Figure 2)

The CAR incorporated at the surface of T cells has a T cell receptor (TCR)-like structure. The extracellular part consists of a single variable chain fragment (scFv) of an antibody that recognizes the antigen present on the cancer cell surface. The transmembrane and intracellular parts contain co-stimulatory domains that permit the amplification of the signaling and, thus, the response of the T cell following binding to the tumor antigen. In the first-generation CAR T cell design, CD3ζ was the only signaling domain used, but in the following generations, CD28 and/or 4-1BB (CD137) co-stimulatory domains were added to this structure. The choice of these domains is also important for the metabolic status and the survival of the CAR T cells. It was observed in patients that the use of 4-1BB permitted a persistence of CAR T cells in time, without exhaustion of those CAR T cells, via the stimulation of the noncanonical nuclear factor kappa B (NF-κB) pathway [69]. In contrast, the CD28 costimulatory domain does not permit the cells to survive longer than 30 days in the patients [70,71,72,73]. This phenomenon can be explained by the fact that the 4-1BB promotes improved mitochondrial function so that T cells can rely on mitochondrial respiration as their energy source, which promotes the survival of central memory T cells by the activation of adenosine monophosphate kinase (AMPK), as demonstrated in mice [74]. When the CD28 co-stimulatory domain was included in the CAR, patient T cells were relying more on glycolysis (via activation of the PI3K/AKT/mTOR axis), which pushed their differentiation towards effector memory T cells [75,76]. Following antigen recognition by the CAR, CD28 stimulated GLUT1 via the PI3K/AKT pathway, linked to the mTOR/Myc pathways, which are also implicated in amino acid/lipid metabolism [17,19]. These data underline the importance of the choice of the co-stimulatory domain to shape CAR T cell metabolism and permit short-term or long-term anti-tumor efficacy. As for CAR T cells, TIL performance also depends strongly on the metabolic status of the malignancy and the TME.

Cytokines also play a role in the metabolic shape of the CAR T cells (see Section 1.3.2). Indeed, the fourth CAR T cell generation, also known as “TRUCKS” (T-cell redirected for unrestricted cytokine-mediated killing), has a transgenic cytokine expression system that improves their expansion and persistence in vivo via metabolic changes [77]. The cytokines employed in this strategy are interleukin 12 (IL12), IL15 and IL18. IL15 can, for example, lower the glycolysis level in human CAR T cells via a decrease in mTOR complex 1 (mTORC1) activity, whilst OXPHOS levels/respiratory capacity and expression of fatty acid oxidation-related genes are increased in these cells. This metabolic phenotype allows the human CAR T cells to have a stem cell memory behavior with higher cell proliferation and in vivo longevity [78]. More recent fifth-generation CAR T cells express a sub-unit of the IL2 receptor (IL2RB) between the stimulatory domains (CD3ζ and CD28/4-1BB). Since IL2RB presents a binding site for STAT3, the cells can activate the JAK-STAT pathway, following the antigen recognition, which leads to a higher proliferation rate. Moreover, these human CAR T cells seem to have a stronger anti-cancer activity against leukemic cells [79].

**Figure 2:**
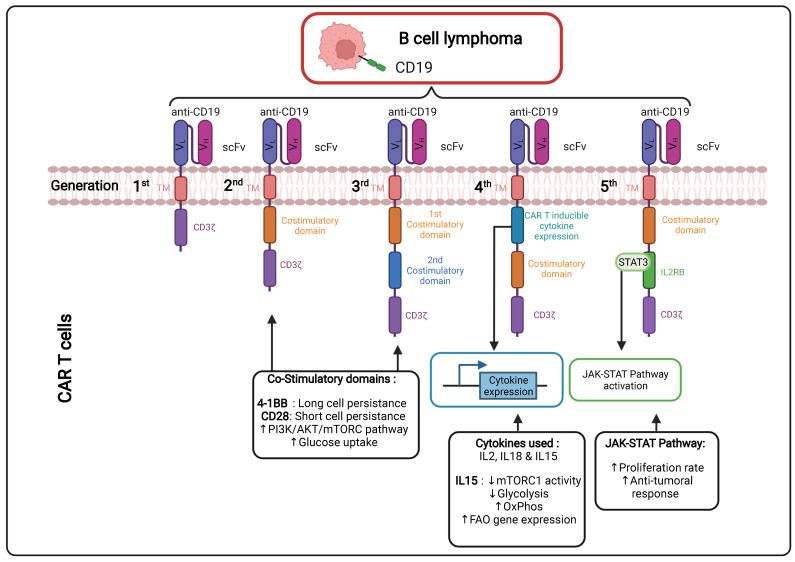
Different generations of CAR T cells and their signaling pathways. TM: Transmembrane; scFv: Single-chain variable Fragment; V_L_: Variable Light chain; V_H_: Variable Heavy chain; IL2RB: interleukin 2 Receptor B; mTORC: mammalian target of rapamycin complex; FAO: Fatty Acid Oxidation; JAK-STAT: Janus Kinase-Signal Transducers and Activators of Transcription; PI3K: Phosphoinositide 3-kinase; Akt: Protein kinase; CART: Chimeric Antigen Receptor T cell. Figure generated.

#### 1.3.2. Ex Vivo Expansion of CAR T Cells to Manipulate Their Metabolism

As they are immune cells and similar to TILs, CAR T cells are highly sensitive to metabolic changes in their environment, which can influence their survival and their functionality. When cultured ex vivo, CAR T cells are often cultured in the presence of supra-physiological nutrient levels in order to generate enough cells for reinfusion. This also means that the cytotoxic efficiency is compromised when changing from an overdosed medium to a nutrient restrictive microenvironment. Thus, the composition of expansion media plays an important role in the process of CAR T cell generation and needs to be optimized in order to facilitate the transition between the ex vivo expansion and in vivo adaptation. For example, carnosine, an amine poorly present in sera, when added to the medium, facilitates human T cell transduction and their in vivo engraftment by switching the main metabolic state from glycolysis to OXPHOS, which allowed a better anti-tumoral response [80]. Currently, expansion media rely essentially either on fetal bovine serum (FBS) or human serum (HS), which does not take into account some blood fractions, which might highly compromise human CAR T cell fitness [81].

The effect of cytokines on T cell metabolism is only beginning to be explored in the field. Survival cytokines, IL2 and IL7, promote glycolysis in T cells [21,82], while IL-15 promotes their mitochondrial biogenesis [36]. TGFβ seems to suppress both glycolysis and mitochondrial respiration. Therefore, the choice of cytokines or growth factors in the media for CAR T cell expansion might be crucial for their in vivo adaptation.

It is therefore important to be aware that the ex vivo expansion of these CAR T cells is a tricky step in the process and can be limiting and compromise treatment efficacy. Several research teams are currently trying to improve CAR T cell therapy by circumventing the ex vivo expansion step and directly generating CAR T cells in vivo. In recent studies, lentiviral vectors specifically recognizing the CD3^+^ or even the CD8^+^ human T cells were used to generate efficiently anti-CD19 CAR T cells directly in vivo and were able to wipe out the targeted healthy and malignant B cells in humanized mouse models [83,84,85,86,87]. Although this still needs to be evaluated, one can speculate that these in vivo generated CAR T cells might expand in their proper environment and maintain their metabolic fitness in vivo.

More recently, an elegant strategy to overcome in vivo CAR T cell exhaustion has been reported with better outcomes than PD-1/PD-L1 blockade, which can improve T cell functions but do not change the epigenetic reprogramming associated with T cell exhaustion. The approach is based on inducing a transient rest in CAR signaling and, therefore, temporally blocking the sustained CAR T cell activation that leads to exhaustion [88]. This approach made use of temporal downregulation of the surface expression of the CAR by inducing its degradation by pharmacological treatments. The tunable control of CAR expression levels not only improved CAR T cell anti-tumoral functions in vivo in terms of intensity and duration but also led to non-exhausted T cells with a memory-like phenotype in xenograft mouse models of leukemia and osteosarcoma. Interestingly, the already established exhaustion of CAR T cells can even be reverted after a transient rest of the CAR signaling [88].

### 1.4. Importance of Nutrients, Metabolites for T/CAR T Cell Metabolism, Survival and Function (Figure 1)

Currently, in the clinic, CAR T cells are essentially used as a therapeutic strategy for hematopoietic malignancies. Unfortunately, the efficiency of this technique is extremely poor to non-existent when it comes to a solid tumor. This can be explained by the low number of known specific antigens in these types of cancers. Furthermore, the TME can, by its composition, affect the metabolic phenotype of these cells, and even a slight change in its composition can alter T cell functionality and survival [89,90], as described in Section 1.1.

#### 1.4.1. Glucose Availability in the Tumor Environment

Glucose is the key element for which T cells and cancer cells compete and is therefore limited in the TME. This is a critical metabolite for energy production via metabolic pathways such as glycolysis and OXPHOS. Indeed, once activated, effector T cells increase their glucose uptake via a higher expression of the glucose transporter GLUT1, which permits higher use of glycolysis compared to OXPHOS. The important consumption of glucose by tumor cells, therefore, favors a decrease in glycolysis in immune cells such as T effector cells, thereby reducing their proliferation and cytokine production [54,61].

Interestingly, a side effect of reduced glucose availability also leads to the apparition of memory T cells with higher OXPHOS respiration in a human context [91]. Therefore, recent investigations have tried to use glucose restriction to enhance CAR T cell persistence and response. Indeed, in a lymphoma mouse model, CD8^+^ effector T cells that underwent a transient glucose restriction displayed a higher cell proliferation and persistence in the blood and induced improved tumor clearance. Moreover, effector cytokines such as IFNγ and granzyme B were released at higher amounts from these cells [92]. Currently, the use of glucose restriction in the process of CAR T development in the clinic might still be risky and needs further investigation. However, it is possible that by substituting glucose with other carbohydrates such as galactose, we might improve CAR T cell generation and production in the future.

Alternatively, Qiu et al. revealed that under glucose restriction, acetate was able to rescue effector T cells functions. They showed that exhausted human and murine T cells could be epigenetically remodeled and reactivated by acetate, and this resulted in enhanced INFγ gene transcription and cytokine production [33]. Therefore, interfering with metabolism using acetate might be of therapeutic benefit.

#### 1.4.2. Lactate, a Side Product of Tumor Cell Glycolysis

Other immunosuppressive metabolites are present in this TME. As tumor cells generally rely on glycolysis, lactate is one of these elements. Glycolysis causes greater amounts of lactate production, which leads to acidification in the TME. This influences the murine T cell effector function in terms of cytolytic capacity and cytokine production [93]. Lactate reduces the anti-tumoral response by impairing NAD+ recycling and therefore blocking the enzymatic reactions, involving glyceraldehyde 3-phosphate dehydrogenase and 3-phosphoglycerate dehydrogenase, which promotes the differentiation of murine T lymphocytes in regulatory T cells (Treg) [94]. Lactate also functions as an oncometabolite, which polarizes macrophages to the M2 type and maintains Treg cells in a low glucose TME [94,95,96].

In order to reduce these high levels of lactate in the TME, Mane et al. also used an inhibitor of the lactate Dehydrogenase (LDH), which, when combined with a CAR T cell therapy, reduced the tumor growth in a murine prostate xenograft cancer model treated with human CAR T cells [97,98]. Of note, human CAR T cells might, in the context of a murine tumor, show lower activity. Lactate induced TME acidification and rendered TILs incapable of IL2 and INFγ production and increased the number of regulatory T cells in humans [99,100]. Unsurprisingly, the inhibition of LDH restored human effector T cell proliferation and function [101]. In another approach, Renner et al. [102] used an inhibitor (diclofenac) of the lactate transporters MCT1 and MCT4 to reduce the efflux of lactate from the tumor into the TME. Due to the diclofenac treatment effector, T cells remained functional and were able to control tumor growth. In addition, this drug, which targets glycolysis, also improved immunotherapy outcomes.

#### 1.4.3. Limited Amino Acid Availability

Glutamine is an important essential metabolite and the most abundant aa in blood. Upon TCR signaling, T cells highly express aa transporters and increase their glutamine uptake. Glutamine is a major fuel for metabolic pathways in active T cells [103]. Therefore, glutamine is an important metabolic element for effector T cells and also for tumor cells. Hence, many cancer cell types over-express ASCT2, the main glutamine transporter, which induces a reduction in the glutamine pool that is available for T cells in the TME. Indeed, targeting glutamine uptake in a human cancer xenograft mouse model resulted in reduced tumor development [104]. Therefore, the availability of glutamine is an important element for anti-tumoral response by promoting T cell proliferation and cytokine production [105]. As expected, Leone et al. [106] demonstrated that glutamine blockade in tumor-bearing mice reduced mitochondrial and glycolytic metabolism in cancer cells, which in turn reduced hypoxia, acidification and nutrient depletion in the TME. In contrast, in effector T cells, glutamine blockade induced upregulation of oxidative metabolism and an activated phenotype with effector function [106]. This differential response upon interference with glutamine metabolism between cancer and T cells in a mouse model might be of therapeutic benefit for tumors but needs further investigation. Unexpectedly, one interesting study showed that the restriction of glutamine metabolism during the TCR stimulation led to reduced exhaustion and increased anti-tumor activity in T cells [107]. More in depth studies are needed to reveal the importance of glutamine in different types of cancers and infiltrating T cells.

Other aa play an important role in the competition between tumor and healthy cells. Tryptophan is an essential aa that is also important for the production of certain molecules required by effector T cells. Contrary to glutamine, its catabolism produces several metabolites through the kynurenine pathway that reduce the TCR response and favor T cell apoptosis in humans as wells as in mice [108]. The IDO enzyme that catalyzes the conversion of tryptophan into kynurenine was found to be upregulated in murine cancer cells [109] and is linked to an inhibition of the glycolytic pathway in T cells, which reduces the anti-tumoral response of effector T cells [110]. Therapeutic inhibition of IDO in cancer might therefore restore T cell function [111].

Arginine is essential for protein synthesis and is also involved in immunometabolism through its metabolites such as Nitrite Oxide and polyamines [112]. Similar to glutamine, a lack of arginine in the TME impaired murine T cell function [113] and activation of human and murine T cells, especially through the decrease in activation markers expression such as CD25/CD28 [114,115]. Indeed, higher levels of arginine have been linked to improved survival of memory T cells and anti-tumoral response [112].

#### 1.4.4. Hypoxia in the TME Has Important Effects on TIL Infiltration and Function

Lactate secretion and high glycolytic activity in the tumor cells are linked to a hostile hypoxic environment in the TME. The importance of oxygen availability for T cells was shown by supplying oxygen to tumor-bearing mice, which led to increased T cell infiltration and improved tumor regression [116,117]. This hypoxic state triggers the transcription factor HIF1α in T cells, which is also stabilized by T cell activation and favors glycolysis by upregulation of GLUT1 expression and some other enzymes and regulators. Hypoxia also induces a higher production and release of reactive oxygen species (ROS) that impair T cell mitochondrial functions and induce T cell exhaustion in mice [118,119]. In order to bypass this problem, recent work in the CAR T cell field showed that it is now possible to generate T cells that will express the CAR only in hypoxic conditions. Hence in normoxia, the CAR is degraded, but once in hypoxic conditions, the CAR is stabilized at the cell surface in a murine solid tumor model, and all the pathways required for an effective anti-tumoral response remain active [120].

In hypoxic tumors, adenosine is a major immunosuppressive factor that limits the function of murine as well as human T cells in the TME via activation of the adenosine A_2A_ receptor (A_2A_R) [121,122]. In this regard, CAR T cells deficient for the A_2A_R were engineered, which became insensitive to high adenosine levels and maintained cytokine production, activation of the JAK-STAT signaling pathway and anti-tumor functions [123].

#### 1.4.5. Cholesterol

Cholesterol uptake by TILs in the TME activates their endoplasmatic reticulum (ER) stress response and the inositol requiring enzyme 1α (IRE-1α) signaling pathway, which induces inhibitory receptor expression on murine and human TILs and, as a consequence, their exhaustion [124]. A new mechanism by which the antitumor response of mouse CD8 T cells can be potentiated through modulating cholesterol metabolism was reported. Inhibiting cholesterol esterification in T cells by genetic ablation or pharmacological inhibition of ACAT1, a key cholesterol esterification enzyme, led to potentiated effector function and enhanced proliferation of CD8 but not CD4 T cells. This is due to the increase in the plasma membrane cholesterol level of CD8 T cells, which causes enhanced T-cell receptor clustering and signaling as well as a more efficient formation of the immunological synapse [125]. Cholesterol metabolism still needs to be further studied in order to explain these effects on anti-cancer T cell response.

#### 1.4.6. Mitochondria in T Lymphocytes Infiltrating the TME

Mitochondria are essential for T cells in terms of energy, biosynthesis of macromolecules in order to sustain their clonal expansion and also for T cell effector function. Once activated, effector T cells undergo dramatic mitochondrial remodeling to sustain their functions [29]. However, T cells lose mitochondrial function and mass when they infiltrate the TME. This process coincides with the upregulation of co-inhibitory checkpoint molecules [42]. Furthermore, this process is accompanied by the repression of the transcriptional co-activator peroxisome proliferator-activated receptor-gamma coactivator 1α (PGC1α) that is essential for mitochondrial biogenesis. In accordance, overexpression of PGC1α rescued intratumoral T cell metabolism and improved T cell effector function in a melanoma mouse model [126]. Therefore, increasing mitochondrial mass and quality may armor T cells to resist the hostile tumor environment.

### 1.5. Immune Checkpoint Molecules and T Cell Metabolism in the TME (Figure 1)

Immune checkpoint molecules (ICM) are co-inhibitory receptors expressed by T cells that are essential for preventing autoimmunity or immunopathologies. However, in the context of tumors, they can tune down anti-tumor responses of T cells. Checkpoint molecules and their ligands are expressed by multiple cells in the TME, which can impact the T cell metabolism and efficiency in a more direct manner. Upon antigen stimulation, costimulatory signals such as CD28 are essential for human and murine T cell activation, glycolysis and mitochondrial activity [17,127]. In contrast, ICM such as PD-1 and CTLA-4 revert this process by switching human T cell metabolism and reducing T cell effector functions [128].

Targeting immune checkpoint receptors such and CTLA-4, PD-1 and PD-1 ligand in blood malignancies has proven to be efficient. However, several patients relapsed, and the efficacy in solid tumors was not as successful. Programmed death-1 (PD-1) is a major regulator of T cell exhaustion; thus, human T cells stimulated with a PD-1 Ligand reduced their glucose uptake and used neither glycolysis nor catabolism of glutamine. Conversely, these cells express a higher rate of Fatty Acid Oxidation and lipolysis [129]. PD-1 signaling is also linked to a reduction in the expression of the proto-oncogene cMyc and reduced activity of the PI3K/Akt/mTOR pathway [130]. However, Chang et al. [61] showed that signaling through PD-L1 in tumor cells promotes glycolysis. Antibody-mediated blockade of PD-L1 reduced tumor glycolysis rate and restored the level of glucose in the TME, and consequently improved anti-cancer T cell effector function [61], which might be potentiated by the metformin-induced reduction in tumor hypoxia [42]. Additionally, it was shown that increasing phosphoenolpyruvate (PEP) levels in tumor-reactive T cells through overexpression of PEP carboxykinase 1 (PCK1) in T cells restored their anti-cancer T cell activity that counteracted the low levels of glucose in the TME [49]. Importantly, PD-1 was shown to inhibit PGC1α, inducing a reduction in glycolysis and loss of mitochondrial mass in TILs. In accordance with this observation, this TIL phenotype was reverted by PGC-1α overexpression [42,126].

In order to overcome this dampening of T cell function through immune checkpoints in the TME, CAR T cells have been engineered, in which inhibitory receptors were removed [131,132,133,134,135,136] or that express costimulatory signals or secrete factors that can re-activate the immune system, such as inhibitors or cytokines. This was demonstrated in mice xenografted with human tumors as also in murine cancer models [137,138]. One of these immune-stimulating cytokines is IL12P70, which was reported to increase CAR T cell activity [139,140,141]. Sachdeva et al. [142] used an elegant procedure to achieve two objectives at once by gene editing of CAR T cells, in which they inserted the IL12P70 expression cassette into the PDCD1 locus (coding for PD-1). In this way, PDCD1 regulatory elements control the secretion of IL12P70, which will only be expressed with the CAR T cells encountering the tumor antigen. This concomitantly led to the abolition of PD-1 expression. These IL12 secreting human CAR T cells knock-out (KO) for PDCD1 increased significantly antitumor activity in a patient-derived xenograft mouse model compared to CAR T cells KO for PDCD1 alone or CAR T cell counterparts [142]. These results might be explained by the controlled IL12P70 secretion [139,140,141,143].

Similar to PD-1, CTLA-4 is also important in the process of tumoral immune escape by inhibiting CD28-costimulation in effector T cells and therefore preventing activation-induced glycolysis [144]. By blocking CTLA-4 mediated signaling, T cell stimulation and metabolism can be reverted to glycolysis again by reviving PI3K/Akt/mTOR signaling and turning T cells back into potent effector cells.

Upon engagement, TIM3, another ICM, leads to reduced glycolysis and GLUT1 expression and might also inhibit glutaminolysis [145], while the ICM, lymphocyte activation gene 3 (LAG3) negatively regulates mitochondrial metabolism [146]. TIM3 and LAG3 are highly expressed by exhausted T cells [147,148,149,150].

Unfortunately, from the clinical point of view, tumor cells stimulate not only these immune blockades but also secrete immunosuppressive cytokines and enzymes. It is for this reason that in certain tumors, an inhibitor of the immune blockade should be accompanied by inhibitors of these pathways [151], as reviewed by Tabana et al. [152]. Therapeutic interference with these metabolic regulatory molecules may affect biosynthesis and epigenetic marks that influence T cell function and fate. This is discussed in detail in the next section.

### 1.6. Epigenetics Influences the Metabolic Response of T and CAR T Cells (Figure 3)

In recent years it has become clear that epigenetic remodeling plays a major role in T cell immunometabolism and differentiation, as shown in a murine context [153,154]. Several types of epigenetic events were revealed: DNA modification (e.g., by methylation), histone modification, non-coding RNA (ncRNA)-associated modifications and chromatin organization/condensation. The different epigenetic modifications in T/CAR T cells and their importance for T cell physiology have recently drawn a lot of attention and initiated a new field of research.

DNA modification and, more precisely, methylation generally occur to silence a gene expression, e.g., effector genes are often methylated in naïve/memory T cells and demethylated in effector T cells. Therefore, DNA methyltransferases (DNMTs) are highly active in exhausted T cells. Their inhibition therefore can lead to a reduction in T cell exhaustion and an increase in less differentiated T cells [155]. Interestingly, glucose restriction by itself, for example, can reduce the expression and the activity of epigenetic enzymes such as the methyl transferase enhancer of zeste homolog 2 (EZH2), leading to reduced cytokine expression and mouse cytotoxic T lymphocyte (CTL) exhaustion [156,157].

Imprinting of the “histone code” is an essential mechanism of gene regulation. The core histone proteins undergo post-translational modification relying on metabolites and cofactors, resulting in epigenetic remodeling of genomic regions in the T cells. Therefore, in CAR T cells, anti-tumoral or pro-tumoral elements might be modulated by epigenetic modification. It is indeed possible to target cell metabolism in order to impact epigenetics. For example, S-adenosylmethionine (SAM), a metabolite synthetized from methionine and a methyl donor, can modulate the methylation of DNA and histones. The upregulation of the methionine transporter SLC43A2 in cancer cells results in TILs deprived of methionine, which in turn results in decreased histone methylation and cytokine production in both murine and human T cells [158]. It is, however, not yet clear whether methionine restriction or supplementation might be of benefit as an anti-cancer treatment [158].

α-ketoglutarate, fuel for the TCA cycle derived from glutamine metabolism, regulates demethylation in aerobic conditions [159,160]. Thus, the use of an α-ketoglutarate-dependent demethylation inhibitor such as S2HG facilitates the apparition of central memory CD8^+^ T cells. Alternatively, inhibition of glutamine metabolism may decrease α-ketoglutarate and, as a result, increase a hypermethylation state of the DNA in murine T cells [106,161].

In a mouse tumor context, the histone acetylation marks differ strongly between effector and exhausted T cells [162]. Metabolites such as acetyl-CoA, propionyl-CoA and succinyl-CoA are involved in this process and lead, for example, to H3K17 acetylation of introns, a characteristic of exhausted T cells [126,163]. Memory and effector T cells show higher histone acetylation at the INFγ promoter compared to naïve T cells and exhausted T cells [43,162]. In accordance with this, providing acetate in a glucose-deprived TME can induce histone acetylation in T cells, thereby restoring their effector function [92].

Conversely, histones deacetylation enhanced T cell effector function and anti-tumoral response. Histone acetylation inhibitors were further used to avoid T cell exhaustion. Thus, nicotinamide adenine dinucleotide (NAD^+^) generated from glycolysis can change the cell fate via its regulation of sirtuins that are NAD^+^-dependent deacetylases [164]. These sirtuins can modulate cellular metabolism. For instance, in human CD8^+^ T cells, some sirtuins can maintain the FOXO1 protein stability that promotes OXPHOS metabolism in resting cells. However, they also can regulate cell proliferation via the deacetylation of p65, a subunit of NF-κB [165,166].

Even high levels of lactate can lead to histone modification, also called “lactylation” that is implicated in metabolic changes in macrophages, but its role in T cells remains to be determined even though it can be triggered by metabolic enzymes, such as lactate dehydrogenase, that play important roles in T cell life [167].

In view of the tight interconnection between metabolism and epigenetic modifications in CD8+ T cells differentiation, effector functions and exhaustion, particularly in the TME, it will be essential to try to therapeutically interfere with these processes, as suggested in a recent review by Van Acker et al. [168].

**Figure 3:**
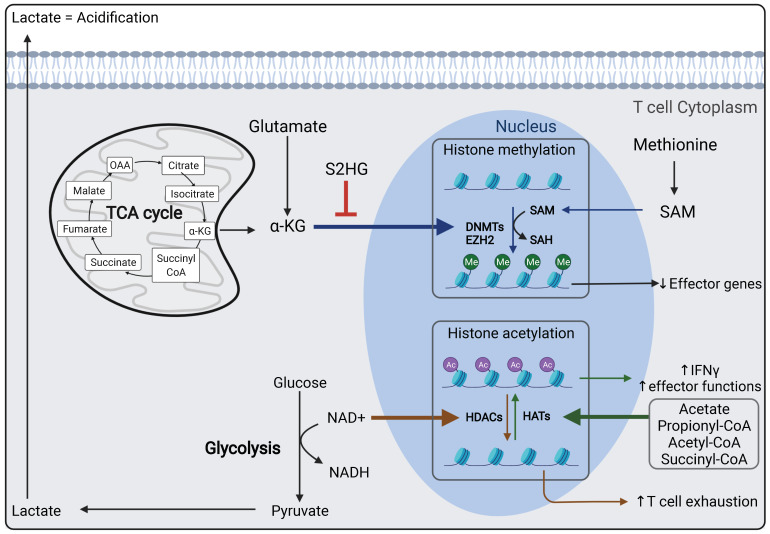
Immunometabolism shapes the epigenome of T cells. Here several of these epigenetic modifying pathways and metabolites influencing histone modification are outlined. OAA: Oxaloacetate; a-KG: a-Ketoglutarate; S2HG: S-2-hydroxyglutarate; DNMTs: DNA methyltransferase; EZH2: Enhancer of zeste homolog 2; SAM: S-adenosyl methionine; SAH: S-adenosyl homocysteine; NAD+: Nicotinamide adenine dinucleotide; NADH: Nicotinamide adenine dinucleotide hydrogen; HDACs: Histone deacetylases; HATs: Histone acetyltransferase. Figure generated by Biorender.com (accessed on 15 November 2021).

## 2. NK and CAR NK Cell-Based Therapies for a First-Line Anti-Cancer Immune Response

In contrast to T lymphocytes, natural killer cells are the granular effector lymphocytes of the innate immune system. Therefore, recognition mechanisms of damaged cells are MHC-independent, and the absence of MHC on any cell elicits the killing program in NK cells. Rather than recognizing specific antigenic peptides on the cell surface, NK cells bind ligands expressed on cells that are infected by viruses, bacteria and parasites or transformed by oncogenes [169]. Once the cytotoxic program is engaged, secretion of cytolytic enzyme-containing granules occurs, as well as production of IFNγ which promotes inflammation. Indeed, NK cells are responsible for a direct and non-primed cell killing, with an early onset during the immune response, orchestrated upon pathogen infection as well as tumor development and progression. Activation of NK cells is the consequence of soluble and membrane-bound signals that come from other immune cells, such as antigen-presenting cells (APCs) and damaged cells. Ligand-binding by a large spectrum of stimulatory and inhibitory receptors on NK cells underlies the self-tolerance and cytotoxic responses displayed by these granular lymphocytes [170]. Even though NK lymphocytes have been classified as part of the innate immune response, memory NK cells have recently been reported [171]. These long-lived memory NK cells and similar NK memory-like phenotypes were described upon infection with human cytomegalovirus (CMV) and upon in vitro treatment with several inflammatory cytokines [170].

### 2.1. NK Cells Display Unique Anti-Cancer Immunosurveillance Mechanisms (Figure 4)

In the context of cancer, NK cells were described to follow two mechanisms for the recognition of oncogenic cellular entities [172,173]. Firstly, by the so-called “self-missing” recognition, malignant cells deprived of MHC (major histocompatibility complex) class I molecules do not signal to the inhibitory receptors, KIRs (killer cell immunoglobulin-like receptors) that are expressed on NK cells, which do not become activated [170,174,175]. The second mechanism relies on the activation of stimulatory receptors on NK cells such as natural cytotoxicity receptors (NCRs) and NKG2D (natural killer group 2D, a C-type lectin-like receptor), which generally bind to heparan sulfate glycosaminoglycans, damage-associated proteins or stress ligands expressed on the surface of cancer cells [176]. Likewise, tumor cells also express ligands binding to immune checkpoint molecules expressed on NK cells, which are in part also expressed on T cells (e.g., PD-1, LAG3, 2B4, T cell immunoreceptor with Ig and ITIM domains (TIGIT)), warranting immune escape from these natural killers [7,177]. As effector lymphocytes, NK cells can kill tumor cells by themselves by directly secreting cytolytic enzymes such as perforin and granzymes. Antibody-dependent cell-mediated cytotoxicity (ADCC) is another mechanism orchestrated by NK cells that directly target tumor cells via CD16 (FcγIIIR) binding to IgG antibodies recognizing tumor-associated antigens (TAAs) [178,179]. Once CD16 expressed on NK cells is engaged, tumor cell killing occurs via secretion of cytolytic granules. At a slower pace but still direct, NK cells can induce tumor cell death once death receptor ligands encounter their cognate receptor (e.g., Fas (CD95) and tumor necrosis factor-related apoptosis-inducing ligand (TRAIL)) on transformed cells. Indirectly, NK cells can secrete a rich repertory of soluble factors including IFNγ, tumor necrosis factor α (TNFα) and granulocyte-macrophage colony-stimulating factor (GM-CSF) that beyond contributing to the establishment of adaptive immune responses dependent on APCs, T and B lymphocytes can induce necrosis of tumor cells [170].

Due to their cytotoxic functions without pre-immunization, these cytotoxic immune cells represent a promising avenue for anti-cancer therapies. For instance, in vivo activation of NK cells using cytokine treatments (e.g., IL2, IL12, IL15, IL18 and IL21) has been investigated in several types of mouse cancer models and human cancers [180,181,182,183]. Furthermore, the use of antibodies to potentiate ADCC as well as to inhibit NK cell immune checkpoints, with similar immunotherapies used for effector T cells activation, are other alternatives that have been tested in mouse cancer models [2]. Antibodies that strengthen ADCC by NK cells include engineered versions that recognize tumor antigens in parallel with a stronger binding to CD16 [184]. In addition, antibodies to activate stimulatory receptors such as 4-1BB in combination with antibodies targeting TAAs are also part of therapeutic strategies under investigation [185]. Direct in vivo activation of NK cells using blocking antibodies against KIRs [186], the inhibitory receptor NKG2A [187] and immune checkpoints have also been tested in vitro as well as in pre-clinical studies and clinical trials of different types of cancer, showing that their potential benefits still need optimization. More sophisticated antibody-based approaches to potentiate in vivo NK cytotoxicity use bi- and tri-specific killer engagers that target TAAs and at the same time bind to NK receptors such as NKG2D, CD16 and IL15 cross-linking moieties [170,188].

Owing to the NK cell contribution to adaptive immune responses and the fact that they do not need to be of autologous origin, NK cells are used for adoptive cell transfer-based anticancer therapies. Sources of either allogeneic or autologous NK cells include peripheral blood and cord blood (CB). However, NK cells can also be derived from hematopoietic stem cells, embryonic stem cells and induced pluripotent stem cells (IPSC). Indeed, autologous, but mainly haploidentical allogeneic NK cells, have been used in clinical trials not only for hematological malignancies [189,190,191,192] and also for solid tumors in patients with recurrent ovarian and breast cancer [193], non-small cell lung carcinoma [194] and digestive cancers [195] as well as metastatic melanoma and renal carcinoma [196]. However, the efficacy of NK cell-based immunotherapy for the treatment of solid cancers is rather poor, as NK cells not only need to expand in vivo following adoptive transfer but also infiltrate the tumoral mass and remain activated despite the immunosuppressive conditions of the TME [197]. NK cells derived from umbilical CB are poorer in cytotoxicity but can be expanded more easily and activated with cytokine treatments. Indeed, stem cell-derived NK cells have already been used in pre-clinical studies and clinical trials of liquid malignancies [198]. Evidently, NK cell lines generated from malignant NK cells represent the most expandable sources of NK cells, with the additional benefit of facilitating genetic engineering. Indeed, genetically engineered NK-92 cells are part of ongoing human clinical trials [172]. The most efficient source of NK cells for adoptive cell transfer in terms of cost, delays, cell expansion, in vivo persistence and anti-cancer cytotoxicity is still under investigation, but the most recent evidence indicates that stem cell-derived NK cells are a promising off-the-shelf alternative for anti-cancer therapies.

**Figure 4:**
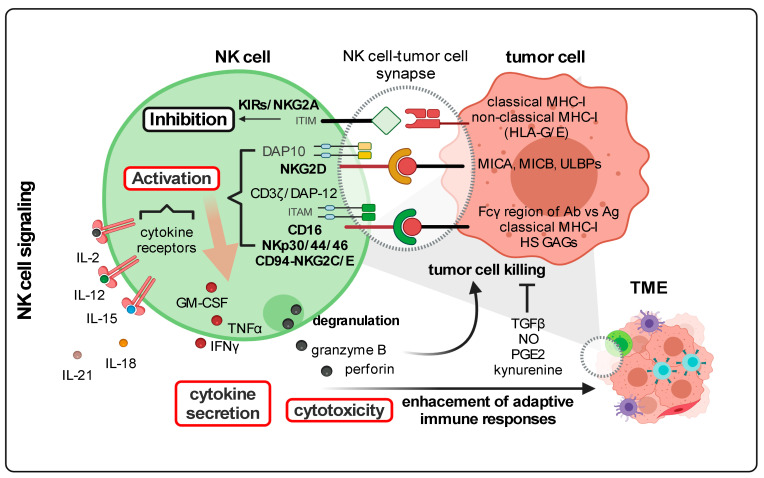
Simplified overview of the human NK cell-tumor cell synapse. Tumor cells signal to inhibitory and activating receptors expressed on NK cells. The binding of KIRs and NKG2A to MHC-I molecules leads to NK cell inhibition; engagement of NKG2D, NKp30/44/46, NKG2C/E and CD16; and drives the activation of NK cells. Upon receptor engagement, inhibitory signaling is mediated by ITIM motifs. Activating signaling can be ITAM-dependent and ITAM-independent. Activating receptors commonly form complexes with adaptor molecules such as DAP10 and DAP12 that trigger the activating signaling cascade. In addition to NK cell activation mediated by receptor engagement, cytokines also stimulate NK cells. Total NK cell activation is characterized by the production of inflammatory cytokines and degranulation, which leads to the release of cytolytic granules containing perforin and granzyme B. Lytic enzymes released at the synapse between NK cells and tumor cells warrant tumor cell clearance. Cytokines secreted by NK cells strengthen adaptive immune responses depending on several soluble factors within the TME such as TGFβ, NO, PGE2 and L-kynurenine that are secreted by tumor and stromal cells, limit the killing of tumor cells by NK cells. KIRs: Killer cell Immunoglobulin-like receptors; DAP: DNAX-activating protein; ITIM: Immunoreceptor tyrosine-based inhibitory motifs; ITAM: Immunoreceptor tyrosine-based activation Motifs; MHC-I: Major Histocompatibility Complex I; HLA: Human Leukocyte Antigen; MICA, MICB: MHC class I chain-related protein A and B; ULBPs: UL16 binding proteins; HS GAGs: Heparan Sulfate Glycosaminoglycans; Ab: Antibody; Ag: Antigen; GM-CSF: Granulocyte monocyte-colony stimulating fFactor; TNFα: Tumor cecrosis factor α; IFNγ: Interferon γ; IL: interleukin; TGFβ: Transforming growth factor β; NO: Nitric oxide; PGE2: Prostaglandin E2; TME: Tumor microenvironment. Figure generated with Biorender.com (accessed on 15 November 2021).

Genetic modifications of NK cells irrespective of their source were performed despite the poor performance of gene delivery achieved into these cells [199]. NK cells lacking CD16 were modified to express this receptor [200], and other NK cell lines were engineered to synthesize IL15 [201]. Furthermore, more specific targeting of tumor cells was pursued by engineering NK cells to recognize TAAs, cellular entities known as CAR NK cells [202,203]. In contrast to T cells that have the property of recognizing TAAs via MHC-I, receptors targeting tumoral antigens are not normally expressed on NK cells. Therefore, engineering CAR for NK cells so that they can recognize specific tumoral antigens is a powerful tool that allows the use of cytotoxic effectors in immunotherapy beyond CAR T cells. Similar to CAR T cells, CAR NK cell design has evolved towards third- and fourth-generation CARs that not only include the CD3ζ chain but also other co-stimulatory molecules such as 4-1BB, DAP-12 and CD28 as part of the activating intracellular domain. The design of the extracellular domain of the CAR in NK cells depends on the type of cancer. For instance, CAR NK cells have already been generated to recognize CD19+/CD20+ B-cell acute lymphoblastic leukemia and chronic lymphocytic leukemia [204,205], CD33+ acute myeloid leukemia [206], CD138+ multiple myeloma [207], CD5+ T cell lymphoma [208], GD2+ neuroblastoma [209], GPA7+ melanoma [210], EGFR+/HER-2+ glioblastoma [211], EpCAM+/HER-2+ breast [201], CD24+/HER-2+ ovarian [212] ROBO-1+ pancreatic [213] and MUC-1+ carcinomas, including colorectal cancer [214].

### 2.2. The Metabolism of Activated NK Cells in a “Healthy” Environment (Figure 4)

Apart from stimulatory and inhibitory signaling at the synapse of NK cells and target cells, the metabolism of NK cells also governs how these granulocytes will proliferate, maturate and function. A low basal metabolic rate, in terms of fluxes through glycolysis and OXPHOS, is a feature of resting or quiescent murine NK cells. For acute NK cell responses, this low metabolic rate is still sufficient for the production of IFNγ in NK cells, upon short-term in vitro stimulation with cytokines or via receptor binding because inhibition of these metabolic pathways limits the production of IFNγ. However, NK cells also participate in immune responses over extended periods, not only displaying cytotoxic functions but also sustaining adaptive immune responses. Prolonged stimulation of murine and human NK cells with different cocktails of cytokines not only activates them but also increases glycolysis and OXPHOS. Augmented glucose uptake and glycolytic rate in activated NK cells are accompanied by increased expression of glucose transporters and glycolytic enzymes [215]. Indeed, upregulation of the glucose transporter GLUT1 was reported in cytokine-stimulated NK cells, which correlated with their increased effector functions, production of IFNγ and granzyme B and NK cell proliferation [216]. Cytokine stimulation was also reported to augment the expression of amino acid transporters [28,197]. Likewise, an augmented OXPHOS in activated NK cells correlates with the higher mitochondrial mass observed in NK effectors during in vivo CMV infection. Upon stimulation, different subtypes of circulating NK cells (CD56^bright^ and CD56^dim^), as well as tissue-resident NK granulocytes, underwent an increase in the energetic metabolism although to different extents [215]. The importance of glycolysis for NK cell effector functions was demonstrated by administrating 2-deoxyglucose (2-DG), a metabolic inhibitor of glycolysis, into mice infected with mouse CMV. Mice treated with 2-DG showed impaired virus clearance and consequently higher viral titers [217].

When active, NK cells rely on glucose to fulfill energetic and biosynthetic demands. Indeed, glucose metabolism and consequently the production of lactate is sustained by pyruvate entering the TCA cycle, which produces citrate in the mitochondria. However, citrate is not further metabolized through the TCA cycle but rather exported to the cytoplasm and converted into malate. This reaction yields NAD+, a cofactor that is needed to sustain glycolysis. In turn, cytosolic malate enters the mitochondria as a carrier of electrons to yield NADH, which fuels the electron transport chain for ATP synthesis. This exchange of mitochondrial citrate by cytosolic malate is known as the citrate–malate shuttle (CMS) and serves to sustain glycolysis as well as the electron transport chain in mitochondria [215]. The glucose dependency of activated NK cells for glycolysis and OXPHOS was reflected by glutaminolysis inhibition, which did not limit OXPHOS nor the effector functions of these granulocytes. Indeed, OXPHOS was shown to be sustained, to a greater extent, by the CMS compared to glutaminolysis in stimulated NK cells [218].

Metabolism of NK cells has been associated with the activity mTOR [219]. mTOR was shown to be involved in the metabolic changes during NK cell maturation but also during the activation of mature NK cells. Similar to T cells, mTOR activity gradually decreased in the transit from the pre-NK to mature NK cell state in order to sustain proliferation and differentiation. Indeed, mature and resting NK cells displayed the lower activity of this master regulator of the cell metabolism. However, activation of NK cells was accompanied by an increase in the activity of mTOR [220]. Indeed, when treated with the classical mTOR inhibitor, rapamycin, mice infected with CMV displayed NK cells with limited proliferative capacity, less production of IFNγ, lower cytotoxicity and consequently higher viral load. Notably, in vitro activated NK cells with different combinations of cytokines or receptor engagement have a variable dependency on mTOR activity to increase glycolysis. Noteworthy, mTOR activity in NK cells is highly dependent on the levels of the amino acids glutamine and leucine [215].

Memory-like NK cells (ML-NK cells) have been described in mice and humans upon infection with CMV as virus-specific effectors that persist after infection [221]. These “adaptive” NK cells are self-renewable and generate pools of NK cells with higher effector functions following a second activation. Interestingly, these so-called adaptive NK cells rely on mitochondrial fitness and OXPHOS to exert an anti-viral and possibly also an anti-cancer program. Indeed, the establishment of mouse memory-like NK cells was reported to depend on the capacity of NK cells to recover a maximal mitochondrial function. This process is achieved upon removal of damaged mitochondria by mitophagy in rapidly proliferating NK cells [215]. Interestingly, this type of NK cells was also described to execute an anti-tumoral killing program with a higher production of INFγ and cytotoxicity and this for a longer period upon reactivation.

The metabolic program of activated NK cells is not only sustained by mTOR but also by other metabolic regulators such as sterol regulatory element-binding proteins (SREBPs) and cMyc. Interestingly, a non-canonical function of SREBPs that is unrelated to the synthesis of fatty acids and cholesterol was described in IL2/ IL12 stimulated NK cells, which rely on glucose to increase their biomass [222]. Increased proliferation, glycolysis and OXPHOS in stimulated NK cells were dependent on SREBPs, as pharmacological inhibition of SREBPs abolished these changes but did not affect mTOR activation. SREBPs transcriptionally regulate the expression of the mitochondrial citrate transporter and cytosolic malate across the mitochondrial membrane and also regulate the expression of the first enzyme, which catalyzes the cytoplasmic conversion of citrate into malate, which is the ATP citrate lyase. Therefore, these two proteins are critical for the citrate–malate shuttle (CMS). By tracing-based metabolomic analysis, the majority of the citrate detected in stimulated NK cells was cytosolic, and therefore generated by the CMS. The importance of SREBPs in mediating NK cell metabolism and cytotoxicity was demonstrated by in vivo inhibition of SREBPs in melanoma tumor-bearing mice upon adoptive NK cell transfer as it abolished their anti-tumoral effect [222].

Inhibition of SREBPs in cytokine-stimulated NK cells reduces NK cell cytotoxicity to a greater extent compared to the inhibition of mTOR with rapamycin, indicating that SREBPs play a unique role in NK function [223]. Indeed, SREBPs control cMyc expression in mouse NK cells stimulated with IL2 and IL12 as inhibition of SREBPs decreased the protein expression of cMyc. cMyc is known to control the transcriptional expression of the rate-limiting enzyme of de novo polyamine synthesis, ornithine decarboxylase (ODC1). Upon inhibition of SREBPs, ODC1 transcript levels were decreased as well as several polyamines in cytokine-activated NK cells. Inhibition of de novo polyamine synthesis resulted in lower proliferation, glycolytic and OXPHOS rates and less production of IFNγ and granzyme B. However, the activation of NK cells with polyamine addition was not able to rescue the metabolic and cytotoxic defects in NK cells that are caused by SREBP inhibition. Instead, this rescue occurred via the production of IFNγ and granzyme B. Polyamines participate in a post-translational modification known as hypusination that occurs in a translation initiation factor. Inhibition of SREBPs was shown to decrease the transcript levels of one of the enzymes involved in hypusination. Inhibition of hypusination decreased NK cell proliferation, OXPHOS rate, production of cytokines as well as NK cell-mediated cytotoxicity. Therefore, as part of SREBPs and cMyc downstream signaling and apart from SREBP regulation of the energetic metabolism, de novo polyamine synthesis and hypusination seem to be downstream pathways that contribute to the optimal performance of cytokine-stimulated NK cells [223].

Upon stimulation with IL2 and IL12, cMyc, an anabolic transcription factor, was upregulated at the transcript and protein levels in IL15-expanded splenic mouse NK cells. However, the expression of hypoxia-inducible factor-1α (HIF-1α) was not critically modulated by cytokine stimulation. NK cell stimulation increased the expression of the transferrin receptor CD71, glycolytic and OXPHOS rates, as well as mitochondrial mass in a cMyc-dependent and a HIF-1α-independent manner. These metabolic responses in stimulated NK cells were accompanied by increased production of IFNγ and granzyme B. Interestingly, initial cMyc upregulation upon stimulation with IL2 and IL12 was mTOR-dependent for cytokine stimulation less than 18 h and fully independent of Akt-driven signaling. Blocking the highly expressed aa transporter SLC7A5 reduced the expression of cMyc, mTOR activity, energetic metabolism and cytokine production in activated NK cells. While this aa antiporter extrudes mostly glutamine for uptake of some other amino acids such as leucine, only glutamine availability controlled cMyc expression in stimulated NK cells independently of mTOR activity. Therefore, cMyc expression in NK cells was dependent on the SLC7A5 transporter and glutamine, pointing out that high expression of cMyc requires not only rapid translation machinery but also sufficiently available aa. Indeed, the role of glutamine in NK cell responses was shown to be independent of fueling the TCA cycle for OXPHOS, which was consistent with the reliance of activated NK cells on the CMS. Glutamine was shown to be critical not as an energetic fuel but as an aa that is needed for the upregulation of cMyc. In this regard, glutamine deprivation but not inhibition of glutaminolysis dramatically reduced OXPHOS and glycolytic rates, as well as cytokine production and in vitro cytotoxicity of NK cells against tumor cells [218]. This is not exactly the same for T cells residing in the TME (see point 1.2.3)

### 2.3. NK Cells Are Metabolically Challenged within the TME (Figure 5)

Similar to other stromal cells infiltrating the TME of solid malignancies, also equivalent to T cells, NK cells experience metabolic challenges that could impair their cytotoxic functions. For example, glucose restriction occurs due to sugar-devouring tumor cells. Indeed, the TME should be viewed as an area with specific cellular entities, nutrient and oxygen availability, and soluble factors that will shape the functions of already resident and newly incoming cells. For instance, some of the immunosuppressive signals from tumor and stromal cells exert metabolic changes in NK cells that induce their exhaustion. NK cell exhaustion could be detrimental to controlling tumor progression. One example includes the combination of TGFβ with IL2 that not only downregulates the expression of several activating receptors on NK cells but also decreases OXPHOS in activated NK cells, thereby reducing their proliferation and cytotoxic responses [197,224]. TGFβ was reported to limit NK cell metabolism by inhibiting mTOR [225]. Sources of TGFβ within the TME include regulatory T lymphocytes (Treg), M2 macrophages, cancer-associated fibroblasts (CAF) and tumor cells. For instance, TGFβ-secreting Treg cells negatively impact NK cells via the activation of the NK receptor CD69.

**Figure 5:**
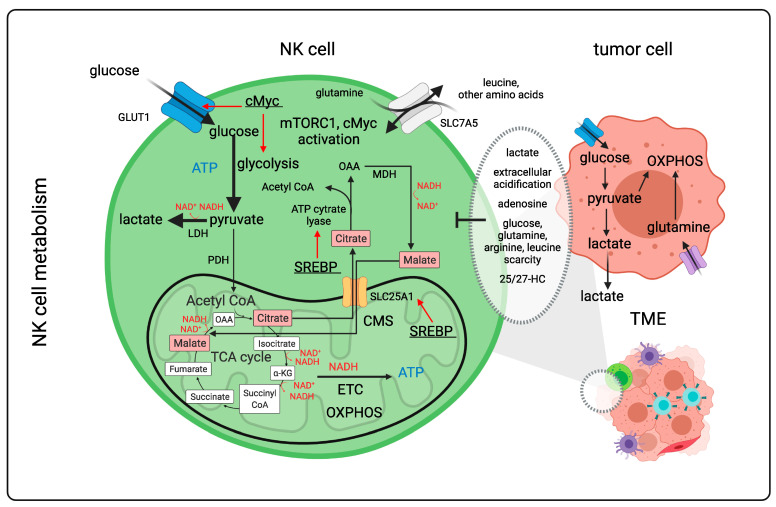
NK cell metabolism upon activation and challenges within the TME. NK cells rely on glucose to sustain glycolysis and OXPHOS for the production of energy. The citrate–malate shuttle between mitochondrial citrate and cytosolic malate provides sufficient reducing equivalents in the cytoplasm to sustain glycolysis and glycolytic ATP production while generating lactate. The citrate–malate shuttle also generates NADH molecules in the mitochondria apart from the ones that are generated by the tricarboxylic acid during OXPHOS. NADH molecules are oxidized by the electron transport chain to produce ATP. Upon NK cell activation, SREBPs transcriptionally controls the expression of the malate–citrate antiporter SLC25A1 and the ATP citrate lyase. In activated NK cells, the glucose transporter Glut1 is upregulated as well as the aa transporter SLC7A5. The exchange of intracellular glutamine for other aa, such as leucine through SLC7A5, increases the intracellular aa availability that is required to activate mTORC1 and enhance cMyc expression. cMyc transcriptionally controls glycolysis as well as the expression of the glucose transporter GLUT1. NK cells at the synapse with tumor cells and within the tumor microenvironment encounter extracellular acidification. This is mediated by a higher content of lactate secreted by tumor cells, inhibitory metabolites such as adenosine, limited availability of glucose and aa such as glutamine, arginine and leucine, as well as soluble inhibitors of SREBPs such as 25/27-HC. LDH, lactate dehydrogenase; PHD, pyruvate dehydrogenase; TCA, tricarboxylic acid; OAA, oxalacetate; α-KG, α-ketoglutarate; ETC, electron transport chain; OXPHOS, oxidative phosphorylation; CMS, citrate–malate shuttle; SREBPs, sterol regulatory element-binding proteins; mTORC1, mammalian target of rapamycin complex 1; MDH, malate dehydrogenase; 25/27-HC, hydroxycholesterol; TME, tumor microenvironment. aa, amino acid. Figure generated by Biorender.com (accessed on 15 November 2021).

#### 2.3.1. The Hypoxic Tumor Environment and NK Cell Function

Several metabolic aspects of the TME were shown to alter NK cell functions. For example, higher levels of adenosine, as a tumor-derived metabolite produced by ATP and AMP that are released by tumor cells in a hypoxic microenvironment, limited NK cell functions similar to T cell functions when adenosine was bound to adenosine receptors expressed on NK cells. Adenosine was reported to decrease OXPHOS and glycolysis of human NK cells, stimulated with IL2 plus IL15, which reduced their cytotoxicity [226]. A hypoxic TME was also suggested to shape NK cell metabolism. Some studies showed that hypoxia could reduce but not entirely abolish NK cell functions. Activation of HIF-1α mediates the transcriptional regulation of glycolytic genes, which might be critical for NK cells in order to keep their cytotoxic effects in a low oxygen environment. However, evidence is controversial since hypoxia also decreased the expression of several activating NK cell receptors such as NKp30, NKp46 and NKG2D [197]. Moreover, a hypoxic TME leads to granzyme B degradation in NK cells through autophagy [224].

#### 2.3.2. Effect of the Metabolite Lactate and Other Metabolites on NK Cell Performance

Our understanding of NK cell metabolism has mainly progressed in the past ten years since immunometabolism was mostly studied in T lymphocytes and myeloid cells [8]. Lactate is one of the metabolites that is readily generated by tumor cells and was shown to blunt the anticancer immune surveillance of NK cells and of T cells, as mentioned earlier. Indeed, local secretion of lactate by tumor cells within the TME of murine melanoma and pancreatic adenocarcinoma models was associated with less infiltration of NK cells, which produced lower levels of IFNγ and granzyme B. In vitro stimulated NK cells did not only secrete lower levels of IFNγ but also reduced levels of IFNγ transcripts when treated with lactic acid or cultured in low pH conditions. Upregulation of nuclear factor of activated T cells (NFAT), a transcription factor controlling IFNγ expression during NK cell activation was also found to be inhibited upon lactic acid treatment and led to apoptosis of NK cells. Extracellular lactic acid was shown to be taken up by NK cells, which caused intracellular acidification and a drop in ATP levels. Therefore, immune cell evasion in these cancer models was dependent on the levels of expression of lactate dehydrogenase A (LDHA) in tumor cells and the levels of lactate within the TME, which dampened the cytotoxic effects of NK cells [53]. Another study in colorectal carcinoma patients found that when the carcinoma metastasized to the liver, lactate was produced, which in turn causes intra-cellular acidification of intra-tumoral NK cells. Additionally, NK cells residing in the liver displayed mitochondrial dysfunction and underwent apoptosis [215].

The NK cell-tumor synapse was described as an energetically demanding connection. Indeed, there is mitochondrial polarization in NK cells to the site of tumor cell docking. This is accompanied by a reduction in the mitochondrial membrane potential of NK cells when the tumor cell is targeted as a reflection of an energy-consuming mechanism [227,228]. Alterations of the glycolytic pathway were described in intra-tumoral NK cells. In a lung cancer mouse model, NK cells displayed a reduced glycolytic rate, which correlated with lower effector functions. Interestingly, fructose-1,6-biphosphatase, an enzyme of gluconeogenesis, was found to be upregulated in these NK cells, in accordance with glycolysis inhibition and an improved NK cell anti-cancer activity upon inhibition of this enzyme [229]. In addition, metabolic alterations can be caused by inhibition of SREBPs, major transcription factors that mediate higher glycolytic rates, cytotoxicity and cytokine production in cytokine-stimulated NK cells. Indeed, higher levels of naturally occurring SREBP inhibitors such as 25- and 27-hydroxycholesterol were found in tumors of patients with breast, gastric or colorectal carcinomas [197]. These compounds are synthesized from cholesterol by enzymes upregulated in macrophages and some tumors [215].

#### 2.3.3. Limited Amino Acid Availability in the TME

Other metabolic challenges encountered by NK cells within the nutrient-deprived TME include reduced aa availability. Although reported in vitro, human NK cell lines and primary cells display lower proliferative capacity and IFNγ production when arginine levels are low. Furthermore, the absence of leucine in the culture media inhibited the mTOR pathway in NK cells. Aside from the aa requirements of NK cells, the production of certain aa byproducts can modulate NK metabolism. In myeloid cells, arginine catabolism that occurred as a result of inducible nitric oxide synthase (iNOS) upregulated the yield of nitric oxide. Nitric oxide in the TME impairs NK cell-mediated ADCC, and the inhibition of iNOS or the depletion of myeloid-derived suppressor cells (MDSCs) was shown to revert NK cell cytotoxicity [230]. Furthermore, myeloid cells also upregulated arginase, an enzyme that catabolizes arginine and depletes this aa in the TME. In addition, L-kynurenine, which is a byproduct of tryptophan degradation and is catalyzed by IDO, was described to inhibit the proliferative capacity of NK cells. A similar scenario was reported for T cells in the TME. This also leads to the upregulation of activating NK cell receptors (i.e., NKp46 and NKG2D) and cytokine synthesis [231]. Activated NK cells display high levels of the L-kynurenine transporter across the cell membrane, and these granulocytes are at high risk of undergoing inhibition by this catabolite within the TME [215]. Apart from producing immunosuppressive catabolites, IDO depletes tryptophan in the TME and therefore reduces the availability of this essential aa [8]. Prostaglandin E2 (PGE2) is another metabolite that NK cells can encounter within the TME. PEG2 is synthesized by cyclooxygenases that can be expressed not only in tumor cells but also in tumor-associated macrophages (TAM) and other stromal cells. PGE2 is known as a critical modulator of NK cellular functions. For instance, PGE2, once bound to EP2 and EP4 receptors on NK cells, can decrease the expression of several activating NK cell receptors such as NKp30, NKp44, NKp46 and NKG2D. In TMEs where PGE2 is secreted, NK cell cytotoxicity is impaired. PEG2 blocking was shown to improve NK cell effector functions in a mouse model of metastatic breast cancer. Likewise, a positive outcome when using inhibitors of cyclooxygenase 2 in several solid malignancies was reported [232].

Recently, the effect of glutathione on NK cell cytotoxicity was described. Pharmacologically blocking the formation of epigenetic enzymatic complexes that contain the demethylase LSD1 did not only decrease the viability of NK cells but also OXPHOS respiration, which had a lesser effect on T cells. In correlation, mitophagy with ROS production and reduced glycolysis were also demonstrated. The oxidative stress and impaired viability were rescued by glutathione supplementation, and the cytotoxic effect of NK cells was also partially reinstalled. This occurred upon scaffolding LSD1 inhibition, despite not reversing the impaired energetic metabolism. Therefore, the redox status of NK cells seems to be crucial for their cytotoxicity, even when their energy metabolism is affected. Improvement of the anticancer effector functions of NK cells beyond an optimal energetic metabolism was suggested with nutritional supplementation of glutathione [233].

#### 2.3.4. Targeting the Metabolism in the TME to Re-Establish NK Effector Function

Boosting NK cell effector functions by targeting the metabolism within the TME is a promising avenue for anticancer therapies. However, this is not an easy task when considering that metabolic pathways are not exclusive to tumor cells and that targeting them might also affect stromal cells [234]. Despite this, several strategies were suggested to overcome the metabolic challenges that NK cells encounter when fighting cancer cells in liquid as well as solid malignancies. For example, chemotherapy-induced tumor cell death might diminish the amount of glucose consumed by tumor cells. Consequently, NK cells may benefit from a TME that is less deprived of glucose. Inhibitors of glutaminase, which reduce the entry of glutamine into the TCA cycle in tumor cells, might increase glutamine availability in the TME in order to activate mTOR and induce cMyc translation in NK cells. Treatment with inhibitors of the glycogen synthase kinase 3 (GSK3) in order to avoid cMyc degradation might retain NK cells in their active state. Indeed, GSK3 inhibitors were described to increase the antitumor cytotoxic functions of NK cells [234]. These strategies could also be applied in combination with adoptive NK cell transfer. In the context of CAR NK cells, it is fair to consider whether there are versions of CARs that increase NK cell effector functions by making them more metabolically fit and resistant in order to fight malignant cells.

### 2.4. How Can CAR NK Cells Persist, Remain Cytotoxic and Metabolically Fit in TME?

When considering adoptive cell transfer, CAR NK cells were developed as an alternative to CAR T cell-based therapy, as several side effects can occur when delivering genetically modified T lymphocytes. Some of the limitations associated with CAR T cell immunotherapy include graft-versus-host disease (GVHD), cytokine release syndrome (CRS) and immune cell-associated neurotoxicity (ICAN) [235]. Although not very well established, CAR NK cells are believed to be less prone to causing GVHD because of the strong regulation of their self-tolerance for healthy as well as “self” tissue, which are mediated by inhibitory NK receptors. As part of innate immunity, NK cells do not generate antigen-specific clones as compared to T cells. In addition, NK cells present different properties to elicit the production of myeloid-derived cytokines that might generate an inflammatory storm. Apart from side effects that are minimized with CAR NK cells, these biological entities carry a broad spectrum of activating receptors, which might warrant CAR NK cell activation, even though tumor cells undergo immune-editing and lose the antigen to which the CAR is specific [236]. CAR NK cells as artificial effector lymphocytes can display CAR-dependent and CAR-independent mechanisms of action, a property that makes them valuable for immunotherapy. Indeed, the first clinical trial (NCT03056339) for CD19-targeting CAR NK cells derived from CB in refractory B cell lymphoma patients reported a positive clinical response in more than 50 % of the individuals with no signs of the above-mentioned side effects [237].

Metabolic interventions to improve the immunosurveillance of tumor-infiltrating immune cells have not yet been explored to the same extent compared to targeting tumor cells with antimetabolic drugs. Indeed, targeting metabolism within the TME is another avenue that is currently being investigated in combination with classical immunotherapy but primarily in the context of T cells. For instance, in melanoma, neutralizing the acidic pH of the TME with oral bicarbonate showed improved tumor growth control in combination with anti-PD-1 treatment. Likewise, oral bicarbonate, in combination with adoptive T cell transfer, extended the survival of tumor-bearing mice [238,239]. Other strategies focused on glucose and lactate metabolism in tumor cells [49,240,241,242,243]; however, targeting these metabolic pathways exclusively in malignant cells remains a challenge and is still in progress [80,244,245]. Currently, the following are being investigated and refined: targeting glutamine metabolism in tumor cells [106,246], arginine metabolism in myeloid cells [247], tryptophan metabolism in the TME [248], lipid metabolism in both, tumor [207] and immune cell populations [249] and disruption of signaling pathways activated by nutrient availability and oxygen content (i.e., mTORC1 [250,251], AMPK [252], HIF-1α [234,253,254]). Of note, most of these studies include T or CAR T cells.

When focusing on enhancing the anticancer performance of NK cells beyond CAR engineering in order to target different TAAs and stress ligands, different CAR constructs were designed to improve NK cell-intrinsic activation pathways and trafficking. Likewise, there are CAR versions that intend to reduce the tumor cell heterogeneity within solid TMEs. These CAR versions generate NK cells, which target NKG2D-expressing cancer stem cells and immune checkpoint expressing tumor cells that evade T cell recognition. Furthermore, CAR NK cells were generated to avoid immunosuppressive signals within the tumor, such as TGFβ [214]. How all these CAR constructs contribute to NK cell metabolic fitness remains to be fully interrogated. To our knowledge, genetic modifications that potentiate the metabolism of CAR NK cells have not yet been reported. One major obstacle when using primary NK cells in immunotherapy is the lack of an efficient gene transfer method. Recently, though, two independent studies showed that this hurdle could be overcome by changing the vesicular stomatitis G (VSV-G) envelope glycoprotein (gp) at the surface of a lentiviral vector (LV) by a baboon retroviral envelope gp [255,256]. These new LVs ensured up to 80 % genetic modification of activated NK cells [199,257] and were shown to generate functional CAR-expressing NKs. Another study also showed high-level CAR delivery into NK cells by employing an α-retroviral vector system [258]. These results will pave the way to progress CAR NK cell therapy to the clinic.

**Figure 6:**
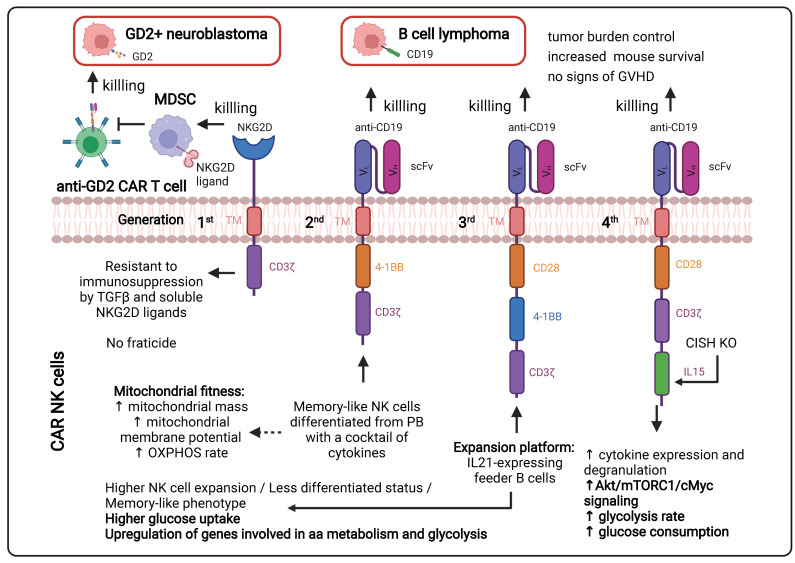
Overview of CAR NK cell generations. Depicted designs are representative of CAR NK cells resistant to challenges within the tumor microenvironment that limit NK cell metabolism and CAR NK cells with reported improvement of metabolic features. MDSCs, myeloid-derived suppressor cells; TGFβ, transforming growth factor β; tCD19, truncated CD19; GVHD, graft-versus-host disease; OXPHOS, oxidative phosphorylation; aa, amino acid. Figure generated by Biorender.com (accessed on 15 November 2021).

In the following section, we illustrate with the few available studies how evaluating the metabolic fitness of CAR NK cells may provide a better in vitro validation of NK cell performance prior to pre-clinical and clinical testing. Much of the knowledge about NK cell dysfunctions is based on pathologies such as obesity. It is very well established that NK cells from obese mice do not respond efficiently to viral infections. This is indicated by fewer cytotoxic responses and a lower number in the circulation, and a number of factors that make these cells less effective against tumors [223]. Immune dysfunction due to obesity has been associated with a lower energetic metabolism in NK cells upon cytokine stimulation. Indeed, lipid accumulation mediated by peroxisome proliferator-activated receptor (PPAR) downregulates cMyc expression, mTOR activity and decreases the rates of glycolysis and OXPHOS in NK cells, which limits their anticancer response [259]. The chronic low-grade inflammation underlying obesity occurs as a result of inflammatory soluble factors secreted by adipocytes and adipocyte-associated macrophages. This inflammatory niche resembles the inflammatory TME of several solid tumors. Local microenvironments with such characteristics induce MDSCs, an immune cell population that is common in oncogenic malignancies and also detected in obese mice driven [260]. Therefore, CAR NK cells were designed to target not only tumor cells but also MDSCs within the TME. MDSC-containing TMEs are often immunosuppressive due to the presence of mediators such as TGFβ that, for instance, downregulates the expression of NKG2D co-adapter molecules on NK cells. A CAR targeting NKG2D ligands was designed to express a fusion protein containing the extracellular domain of the activating NK receptor NKG2D and the CD3ζ chain of cytotoxic T cells as the intracellular domain. NKG2D ligands are not only expressed by damaged and hypoxic tumor cells but also by intra-tumoral MDSCs. This makes NKG2D a suitable receptor for dual targeting in solid malignancies [261]. NKG2D CAR NK cells artificially and constitutively express a functional activating receptor that naturally is usually reduced in the TME. NK cells modified with this CAR displayed higher NKG2D-mediated cytotoxicity in vitro against tumor cells expressing NKG2D ligands. Importantly, this stronger cytotoxicity was retained irrespective of the presence of TGFβ and NKG2D soluble ligands in the cell culture medium, which are components of in vivo TMEs that limit NKG2D signaling. NKG2D CAR NK cells also displayed enhanced cytotoxicity against autologous NK2GD ligand-expressing MDSCs (Figure 6). CAR NK cells were able to limit tumor growth in a xenograft model that was generated by engrafting MDSC and GD2+ neuroblastoma cells, which did not express NKG2D ligands. Importantly, a significant tumor growth control was dependent on the elimination of NKG2D ligand-expressing MDSCs rather than directly targeting tumor cells. These engineered CAR NK cells did not react against NKG2D ligand-expressing autologous T cells as compared to the NKG2D CAR T cells, further demonstrating the safety of the adoptive CAR NK cell-based immunotherapy in terms of GVHD [261].

Memory-like (ML) or adaptive NK cells in humans were reported to display distinctive metabolic features when compared to non-adaptive NK cells. Aside from having an augmented mitochondrial mass, mitochondrial membrane potential and OXPHOS rates, mTOR activation is more enhanced upon CD16 engagement as compared to classical human NK cells in their ML counterparts [215]. This metabolic fitness has not yet been reported as the cause of higher cytotoxicity against tumor cells for longer periods. A recent study, however, clearly shows that ML NK cells were not only suitable for adoptive cell transfer but can also be engineered with a CAR and displayed improved tumor control compared to conventional CAR NK cells. Indeed, engineering ML NK cells derived from PB with a CAR targeting CD19 has shown to improve IFNγ production, degranulation (CD107a+) and cytotoxicity in vitro when co-cultured with NK-cell resistant lymphoma cells as compared to conventional CAR NK cells. This was confirmed in vivo [236].

Other alternatives to expand human NK cells are currently being tested in order to obtain metabolically fit CAR NK cells with stronger cytotoxic responses for clinical application. For instance, expansion of NK cells was performed under co-culture conditions using irradiated feeder B cells that were genetically modified to express membrane-bound IL21 and none or low levels of MHC-I. Expansion in the presence of IL2 and IL15 and a CD19 CAR encoding retroviral vector yielded metabolically fit CAR NK cells. These feeder B cells proved to be advantageous for NK and CD19 CAR NK cell proliferation, purity and reduced their apoptosis. CD19 CAR NK cells expanded using IL21-expressing feeder B cells were more cytotoxic against CD19+ lymphoma cells and displayed higher tumor burden control in two lymphoma xenograft models [262]. CD19 CAR NK cells that were expanded using IL21^+^ feeder B cells upregulated genes involved in aa metabolism and glycolysis, while genes involved in NK cell activation, differentiation and cell-to-cell adhesion were downregulated, reflecting a less differentiated state. Higher glucose uptake was also confirmed in feeder B cell-expanded NK cells. Moreover, genes coding for cell death receptors and cognate ligands were downregulated upon expansion, indicating that these NK cells were less prone to undergo cell death. Furthermore, some transcription factors and signaling proteins that are downregulated in the ML NK cell phenotype were expressed at lower levels when using the feeder B cell expansion system. This highlighted that the resulting NK cells had a potentiated adaptive phenotype that resembled ML NK cells. Furthermore, several metabolic genes such as cMyc, SLC7A5, transferrin receptor (TFRC), serine sulfhydrase (CBS), phosphoserine phosphatase (PSPH), phosphoserine aminotransferase 1 (PSAT1) and genes contributing to the CMS, malate dehydrogenase 1 (MDH1) and pyruvate dehydrogenase (PDHA1) were all upregulated. This study, therefore, described a novel human NK cell expansion system that was superior in generating allogeneic CAR NK cells with an ML NK cell phenotype. This phenotype marked by enriched expression of metabolic genes was associated with rapid cell proliferation without inducing exhaustion. This highlights the feasibility of CAR NK cell-based immunotherapies, as CB banks are readily available [262].

Recently, the fourth generation of CAR NK cells was developed to express an anti-CD19 CAR, specific for B cell lymphoma and IL15 (Figure 6). Moreover, these so-called armored CAR NK cells were further modified to inhibit an NK immune checkpoint. The genetic modification is based on knocking-out *CISH*, a gene coding for the cytokine-inducible SH2-containing protein (CIS). CIS is activated as part of a negative feedback mechanism induced by IL2 and IL15 stimulation [263]. Targeting this cytokine immune checkpoint improved the metabolic fitness of NK cells that were derived from CB. These CAR NK cells, when co-cultured with lymphoma cells, displayed a higher Akt/mTOR/cMyc signaling and glycolysis, which was confirmed by increased extracellular acidification. These IL15 secreting anti-CD19 CAR NK cells were more cytotoxic in vitro and persisted longer in mice when *CISH* was silenced. Adoptive transfer of these *CISH* KO CAR NK cells significantly prolonged the survival of a lymphoma mouse model. This innovative way of specifically improving CAR NK cell immunotherapy is due to the synergism of combining specific tumor cell targeting and cytokine activation by the NK cells themselves while abolishing the cytokine-associated negative feedback. Secretion of IL15 by CAR NK cells gives them a therapeutic advantage since some TMEs are poor in IL15. Importantly, these CAR NK cells are also equipped with a suicide gene that allows inducing apoptosis if adoptive cell transfer results toxic in the clinic [264].

## 3. Perspectives

Currently, the field is investigating the missing links between the proof of concept of innovative strategies in order to overcome current limitations in CAR T/NK cells and their translation into the clinic. Among these hurdles, CAR T/NK cells encounter immune responses, inhibitory signals, metabolic changes from the tumor cells and tumor microenvironment, toxic side-effects and loss of long-term persistence, among others. The field is actively attempting to find solutions to these obstacles by multiple innovative approaches. These approaches include gene editing techniques and in vivo generation of CAR T cells [265] in an attempt to improve the accessibility of the CAR T cell therapy to more patients. In the future, improved mice models that mimic human hematopoiesis and immune response more closely [266] will assist the field with addressing pertinent unanswered questions. Below, we discuss some novel research avenues in the CAR T/NK field.

### 3.1. “Off the Shelf” Universal CAR T Cells

T cell and CAR T cell therapies currently rely on autologous T cell transfer, which requires patient-specific manufacturing. This is a costly process and can lead to heterogeneous products from one patient to another. Therefore, huge efforts are invested into generating allogeneic T cells, which have strong anti-cancer potency that is not rejected by the patient’s immune system. One strategy for universal CAR T cell generation is to eliminate the endogenous TCR, resulting in the sole expression of a CAR on the T cell [131,267,268,269].

Universal anti-CD19 CAR T cells were developed by gene editing strategies to knock out the constant region of the TCRα chain and the CD52 gene, in order to make the CAR T cells resistant to an anti-CD52 antibody that is used for the treatment of B-cell chronic lymphocyte leukemia (Quasim et al. 2017). The treatment of two children with aggressive B-ALL with this universal CAR T was effective. More strategies for the generation of universal CAR T cells as described by Morgan et al. [270].

Mo et al. [271] used an elegant approach based on the fact that recipient T and NK cells, upon allogeneic CAR T cell infusion, may upregulate co-stimulation receptors such as 4-1BB, while resting T cells will not. Therefore, they engineered therapeutic T cells carrying an anti-CD19 CAR together with an allogeneic defense receptor exposing 4-1BB Ligand at their surface, which was shown to eliminate the recipient T cells and NK cells that reacted against this CAR T cell graft. Here, it is obvious that large metabolic changes are involved and that we might need to rewire these universal CAR T cells to make them even more potent and persisting long-term.

Pluripotent stem cells, such as human induced pluripotent stem cells (hiPSCs), can provide an unlimited T cell source for CAR T cell development, with the potential of generating off-the-shelf T cell products. T-iPSCs (iPSC-derived T cells) are phenotypically defined, expandable, easily genetically manipulated and are as functional as their physiological T cell counterparts. The combination of iPSC and CAR technologies provides an exciting opportunity for oncology and greatly facilitates cell-based therapy for cancer patients. However, T-iPSCs, in combination with CARs, are at the early stage of development and need further pre-clinical and clinical investigation (for review, see [272]).

### 3.2. Combining CAR T and CAR NK Cells to Increase Their Anti-Tumor Activity

The most recent studies on CAR NK cells reflect that evaluation of CAR NK cell effectivity for immunotherapy cannot only rely on the assessment of effector functions, including cell killing and production of cytokines. Other parameters such as cell phenotype as well as metabolic features are critical when testing the in vitro efficacy of CAR NK and T cells. Therefore, immunotherapy pursuing to enhance T and NK cell effector functions in parallel with the objective to minimize cancer immune-evasion is beginning to yield promising results [7]. Recently, CAR T and CAR NK cells were combined to pursue this objective. For example, adoptive NKG2D CAR NK cell transfer enhanced tumor infiltration of GD2 CAR T cells that were delivered after CAR NK cell education into a subcutaneous xenograft containing neuroblastoma cells and MDSCs. In contrast to CAR T cells, which demonstrated an impaired infiltration in MDSC-containing tumors in the absence of CAR NK cell education, CAR NK cells homed into the tumor core, irrespective of the presence of MDSCs in the tumoral mass. GD2 CAR T cells were also able to limit tumor growth and increase survival in the presence of MDSCs in mice educated with CAR NK cells. Therefore, NKG2D CAR NK cells that did not directly target neuroblastoma cells due to their lack of NKG2D ligand contributed to limiting tumor growth by eliminating NKG2D+ MDSCs and increasing infiltration of neuroblastoma-targeting CAR T cells [261]. Therefore, this study demonstrated the synergism of combining CAR T and NK cell therapy for improved control of tumor progression.

### 3.3. NKT Cells, the Natural Hybrids of T and NK Cells at the Cutting Edge of CAR Therapies

The potential of combining T and NK adoptive cell transfer as immunotherapy might rely on the mechanisms of malignant cell recognition by T and NK cells. Whereas higher expression of MHC-I leads to stronger activation of T cells, the presence of MHC-I molecules inhibits NK cell targeting. Indeed, natural killer T cells (NKT cells) at the border of T and NK lymphocytes represent an immune population with mixed mechanisms for target cell recognition. NKT cells constitute a subgroup of T lymphocytes, which expresses some NK cell markers. Similar to T cells, NKT cells express a TCR, which is restricted to glycolipid antigens and not peptides. Glycolipid antigens are presented on CD1 molecules, which are MHC-like and expressed by hepatocytes, adipocytes and professional APCs that present microbial antigens to T lymphocytes [177,273].

NKT cells were shown to possess higher glycolytic capacity compared to T lymphocytes upon TCR engagement [177]. These so-called “innate-like T lymphocytes” are activated by T cell-like mechanisms via TCR engagement. In parallel, an active or inhibited state also depends on the engagement of the NK cell receptors expressed on NKT cells. Indeed, activation via TCR binding to glycolipids presented by CD1 was suggested to be interrupted by KIR ligation. Although NKT cells display the capacity to kill tumor cells in a CD1-dependent manner, the role of NKT cells in vivo has mainly been described as regulatory. The secretion of a repertory of cytokines by NKT cells positively or negatively contributes to T and NK cell generation and activation [273]. NKT cells with a morphology resembling NK cells because of their granularity and a low nucleus-to-cytoplasm ratio combine unique characteristics of T and NK cells. However, low numbers, anergy and the NKT cell switch from Th1- to an immunosuppressive Th2-like phenotype were observed in cancer patients. Therefore, injection of the activating glycosphingolipid α-galactosylceramide was attempted as immunotherapy. Likewise, adoptive cell transfer of autologous ex vivo activated NKT cells was studied in clinical trials. CAR NKT cells targeting different oncogenic malignancies were also tested in pre-clinical models [274]. These attempts did not result in high efficacy or preventing and reversing anergy, and maintenance of the Th1-like phenotype has therefore been suggested to boost NKT tumor killing [274]. These attempts did not result in high efficacy; therefore, preventing and reversing anergy and maintenance of the Th1-like phenotype were suggested to boost the NKT tumor killing [273].

Additionally, CAR primary NKT cells were designed to target GD2 ganglioside-positive neuroblastoma cells. The intracellular CAR domain included the CD3ζ chain, CD28 and 4-1BB sequences. The GD2 CAR NKT cells were more persistent than unmodified NKT cells in a metastatic neuroblastoma xenograft model. In addition, serum levels of IFNγ and GM-CSF post-adoptive cell transfer were higher, indicating that the CAR design was effective in polarizing NKT cells into the Th1-phenotype. Furthermore, when adoptively and repeatedly transferred to immunodeficient mice with developing neuroblastoma metastasis, these CAR NKT cells not only delayed the metastatic growth but also significantly increased animal survival. Indeed, CAR NKT cells had the advantage to infiltrate neuroblastoma tissues and displayed no signs of GVHD, compared to CAR T cells [274]. In addition, NKT cells were shown to co-localize in tumors with TAMs. Indeed, NKT cells have the potential to target glycolipid-expressing TAMs via CD1 recognition. Therefore, CAR NKT cells are not only specific for tumor cells but can also shape the TME by making it less immunosuppressive. A more advanced version of this CAR was engineered to express IL15. These GD2.CD28.IL15 CAR NKT cells that target neuroblastoma indicated encouraging results in pre-clinical cancer models and were chosen for an initial clinical trial. In a pre-clinical setup, this design improved in vivo persistence and tumor infiltration, as well as limiting to a greater extent the tumor growth, compared to GD2 CAR NKT cells that do not express IL15 [275]. Furthermore, CD19 CAR NKT cells were also generated and tested in a B lymphoma mouse model. CAR design, including CD62L, was successful in generating CAR NKT cells that were able to control B cell lymphoma growth [176]. However, how we can metabolically interfere with this in order to increase the CAR NKT potency in vivo is currently unknown.

## 4. Conclusions

One of the major advantages of CAR NK over CAR T cells consists in the fact that NK cells can be used as an off-the-shelf product, while CAR T cells can only be used in an autologous setting in order to avoid GVHD. Currently, the generation of CAR T cells though is slow and very expensive. For this reason, universal CAR T cells using gene-editing technologies [270] are under development in order to allow their use in an allogeneic setting. However, in the TME, both NK cells and T cells encounter similar metabolic challenges but also distinct ones that impede their anti-cancer function. These similar or distinct general and metabolic features for both NK and T cells are listed in Table 1, in which we made a side-by-side comparison, including metabolic intervention strategies that are currently explored. Further identifying key cellular and molecular mechanisms that regulate NK and T cell metabolism will reveal new and exciting strategies to engineer innovative CAR NK and T cells in order to overcome the immunosuppressive TME and promote longevity and metabolic and functional fitness.

In summary, multiple strategies to improve the metabolic fitness of CAR T and CAR NK cells in the tumor environment are being explored. Moreover, the first results demonstrated that it might be useful to combine both cell types as described above to overcome the hurdles in the hostile tumors and TME. Even if improved off-the-shelf CAR-expressing T and NK cells reduced the costs of this cell-based immunotherapy, in the future, it would be of vital importance to generate metabolically fit CAR NK and T cells directly in vivo [265].

## Figures and Tables

**Table 1:** Comparison between T and NK cell recognition mechanisms, metabolic features, approaches to improve effectivity of adoptive cell transfer, CAR designs boosting their metabolism and strategies to improve metabolic fitness of CAR-expressing cells.

Features	Active Effector T Cell	Active Effector NK Cell
Immune cell activation mechanisms	TCR engagement via recognition of peptides onto MHC-I in target cells [7]	No requirement of MHC-I on target cells, activation through stimulatory receptors [172]
Energetic metabolism	aerobic glycolysis and OXPHOS via the TCA cycle [7,20].	aerobic glycolysis and OXPHOS via the CMS [215]
Metabolic phenotype	glycolytic [7]	glycolytic [215]
Energetic sources	glucose and glutamine [20]	glucose [215]
Metabolic regulators	PI3K/Akt/mTORC1 pathway cMyc, HIF-1α, glutamine [7]	mTORC1 dependent on and independent of PI3K/Akt pathway cMyc, SREBP, glutamine [215]
Metabolism of memory (-like) phenotype	OXPHOS [34]	OXPHOS [215]
Metabolic approaches to enhance immune cell metabolism, effector functions and persistence upon adoptive cell transfer	In vivo inhibition of the lactate transporters MCT1 and MCT4 by diclofenac in a melanoma mouse model renders tumors sensible to PD1 blockade [102]	Pharmacological inhibition of SREBPs in a melanoma mouse model controls tumor burden [222]
	Glucose restriction for expansion of CD8 T+ cells prior to adoptive transfer into a lymphoma mouse model drives better tumor burden control [92]	Ex vivo pharmacological inhibition of fructose-1,6-biphosphatase in infiltrating NK cells from lung tumors in mice enhances glycolysis in vitro and in vivo tumor control upon adoptive cell transfer [229]
	In vitro and ex vivo administration of acetate in glucose-restricted CD8+ T cells and exhausted T cells, respectively, increases cytokine expression. Silencing of the acetyl-CoA synthetase controls better the tumor burden of a lymphoma mouse model [33]	
	Overexpression of PEP carboxykinase 1 [49] and PGC1α [42] in T cells transferred into melanoma-bearing mice lead to higher tumor cytotoxicity.	Pharmacological inhibition of GSK3 in NK cells from PB expanded with IL15 increases maturation and tumor cytotoxicity in mouse model of ovarian cancer [276]
	Oral bicarbonate in tumor-bearing mice controls tumor growth upon PD1 and/or CTL4 blockade and upon adoptive T cell transfer in melanoma-bearing mice [234]	
Advantages of adoptive CAR-expressing cell transfer as a therapy	Commercial approval of several CAR T cell therapies by the FDA [277] T cells are more suitable for bioengineering by classical viral vector transduction [257]	No need for cells of autologous origin [172] Less prone to GVHD [172]
CAR designs and metabolic fitness	4-1BB-containing CAR: OXPHOS metabolism [75] and longer in vivo persistence [72] CD28-containing CAR: glycolytic metabolism [75] and shorter in vivo persistence [72]	NKG2D-expressing CAR resistant to the immune and metabolic suppressor TGFβ drives MDSCs clearance and better tumor burden control of CAR T cells targeting neuroblastoma in mice [261].
	Hypoxia-inducible CAR expression for better tumor control in mouse models of ovarian cancer and neck and head cancer [120]	IL15-expressing CAR increases in vivo persistence and survival of a lymphoma mouse model [278]
Metabolic strategies to improve fitness of CAR-expressing cells in the TME	IL15 stimulation of CAR T cells reduces glycolysis, increases OXPHOS and FAO genes and leads to a stem cell memory phenotype, high proliferation, longer in vivo persistence, tumor burden control and survival of a lymphoma model [78]	Cytokine-induced memory-like (ML) NK cells modified with a CAR displayed better tumor burden control in lymphoma mouse models as compared to conventional CAR NK cells and ML NK cells [236]
	LDH depletion in prostate tumors improved cancer growth control by CAR T cells [98]	Genetic deletion of the IL15 immune checkpoint in IL15-expressing CAR NK cells increases mTOR and cMyc pathways, glycolytic rates and survival of a lymphoma model [264]
	A_2A_R deficiency in mouse and human CAR T cells improved tumor burden control in breast and ovarian cancer mouse models, respectively [123]	NK cell expansion with IL21-expressing feeder cells increases the expression of several metabolic genes, glucose uptake and promotes a less differentiated phenotype while enhancing tumor cytotoxicity in lymphoma mouse models [262]
	PD1 silencing and expression of IL12 in PD1 deficient CAR T cells increased survival of a lymphoma xenograft mouse model [142]	
